# Conserved neuronal-like and secretory programs define the spatial architecture of gastroenteropancreatic neuroendocrine tumors

**DOI:** 10.1172/JCI204119

**Published:** 2026-09-01

**Authors:** Julie Karam, Samantha E. Hoffman, Amanda Garza, Dan Gui, Hannah I. Hoffman, Breanna M. Titchen, Yutaro Tanaka, Erica Pimenta, Theodora Pappa, Laura Valderrabano, Kevin Bi, Riaz Gillani, Lauren Brais, Erin Shannon, Jason L. Hornick, Jihye Park, Jennifer Chan, Eliezer M. Van Allen

**Affiliations:** 1 Department of Medical Oncology, Dana-Farber Cancer Institute, Boston, Massachusetts, USA.; 2 Broad Institute of Harvard and MIT, Cambridge, Massachusetts, USA.; 3 Harvard/MIT MD-PhD Program, Boston, Massachusetts, USA.; 4 Division of Medical Sciences PhD Program in Biological and Biomedical Sciences, Harvard University, Cambridge, Massachusetts, USA.; 5 Department of Pediatric Oncology, Dana-Farber Cancer Institute, Boston, Massachusetts, USA.; 6 Division of Endocrinology, Diabetes and Metabolism, Mass General Brigham, Boston, Massachusetts, USA.; 7 Division of Hematology/Oncology, Boston Children’s Hospital, Boston, Massachusetts, USA.; 8 Department of Pathology, Brigham and Women’s Hospital, Boston, Massachusetts, USA.

**Keywords:** Genetics, Oncology, Cancer, Macrophages, Molecular genetics

## Abstract

Gastroenteropancreatic neuroendocrine tumors (GEP-NETs) are clinically heterogeneous malignancies whose biology and microenvironmental organization remain poorly understood. Here, we integrated single-nucleus multiomic and spatial transcriptomic profiling across 38 well-differentiated pancreatic (PanNET) and small-intestinal (siNET) tumors to define conserved malignant programs, their regulatory circuits, and spatial niches. We observed 2 conserved malignant cell programs spanning a continuous transcriptional spectrum: a neuronal-like program and a secretory neuroendocrine program. Matched chromatin accessibility profiles uncovered distinct, tissue-specific regulatory networks, including *MAX:MYC* and *MITF* transcription factor binding motifs in siNETs versus *ISL1* and *TFAP4* in PanNETs, indicating organ-specific epigenetic control. Spatial transcriptomic analyses revealed that neuronal-like–high regions localized to densely cellular tumor areas with relative depletion of stromal infiltration, whereas secretory neuroendocrine–high regions occupied fibrovascular and stromal niches enriched for endothelial, fibroblast, and myeloid populations and associated with *TGFB1-ITGB1*, *VEGFA-FLT1*, and *LAMA2-ITGA1* signaling. Across both tumor types, the cNMF2 program was enriched in metastatic lesions and was enriched for profibrotic and proangiogenic gene signatures. Thus, GEP-NETs are organized along a conserved neuronal-to-secretory axis defined by distinct epigenetic programs and spatially coupled to specific microenvironmental niches. This framework unifies NET heterogeneity across organ sites and identifies pathway-specific, microenvironment-linked vulnerabilities for therapeutic targeting.

## Introduction

Well-differentiated neuroendocrine tumors (NETs) are rare malignancies that arise from secretory cells of the neuroendocrine system and can affect multiple organ sites. Gastroenteropancreatic NETs (GEP-NETs), which originate in the gastrointestinal tract or pancreas, account for nearly two-thirds of all NET diagnoses (1). Their incidence has risen nearly 5-fold in the United States over the past several decades, a trend attributed in part to advances in imaging and endoscopy but also reflecting the increasing prevalence of disease in an aging population (2). As a result, solving the clinical challenges posed by GEP-NETs is therefore an increasingly urgent priority.

Despite the increasing incidence, therapeutic advances for GEP-NETs have lagged. Surgical resection remains the only curative option for localized disease, whereas medical therapies typically slow progression without inducing durable remission (3). A major barrier to therapeutic development is the incomplete understanding of GEP-NET biology compared with other gastrointestinal cancers (4). GEP-NETs’ indolent growth has hampered the generation of representative preclinical models, and their rarity has limited the size of clinical cohorts and available samples for characterization. Direct high-resolution profiling of primary human samples therefore represents a powerful strategy to overcome these limitations and reveal new insights into GEP-NET pathophysiology.

Recent single-cell transcriptomic studies of GEP-NETs and their microenvironment have identified immunosuppressive myeloid populations with therapeutic potential or tumor-intrinsic malignant programs (5–7), though the small cohort sizes,heterogeneous sequencing approaches, and incomplete assessment of the microenvironment of these studies have limited generalizability. The spatial architecture of GEP-NETs, particularly their prominent fibrosis and vascular remodeling that may indicate novel biological underpinnings and therapeutic opportunities, has also not been comprehensively examined (8). Additionally, their diverse clinical behaviors suggest malignant cell transcriptional or epigenetic heterogeneity that may underlie subtleties in histology and have clinical relevance.

Here, we hypothesized that integrating single-cell multiomics with spatial transcriptomic profiling of pancreatic and small-intestinal NETs (PanNETs and siNETs, respectively) could elucidate previously unappreciated heterogeneity of malignant cell states and define their interactions with the surrounding stroma and immune microenvironment. Single-cell and spatial technologies now allow dissection of tumor ecosystems at unprecedented resolution, and their application to NETs offers the opportunity to uncover conserved transcriptional programs, epigenetic regulatory networks, and spatial niches that underlie progression and metastasis.

To this end, we present the largest multimodal study of GEP-NETs to date, combining single-nucleus and spatial transcriptomic approaches to define conserved transcriptional programs across samples and tumor types and to uncover their epigenetic regulators, spatial organization, and microenvironmental interactions that may have biological and clinical significance.

## Results

### Multiomic analysis of PanNETs and siNETs at single-nucleus resolution.

We performed joint single-nucleus RNA-sequencing (snRNA-seq) and assay for transposase-accessible chromatin sequencing (snATAC-seq) on 38 archival flash-frozen tumor samples of well-differentiated NETs originally resected at Brigham and Women’s Hospital between 2015 and 2021, consisting of 21 PanNETs and 17 siNETs (Figure 1A; see Methods). In addition, high-quality FFPE slides from PanNET and siNET samples were used to generate spatial transcriptomic sequencing data (*n* = 19) using the 10x Visium platform (Figure 1A; see Methods).

Twenty-five of the samples in our cohort were primary tumors resected from either the pancreas or small intestine. Of the remaining samples, 5 were regional metastatic siNETs, and 7 were distant liver metastases originating from either PanNETs (*n* = 3) or siNETs (*n* = 4) (Figure 1B and Supplemental Table 1; supplemental material available online with this article; https://doi.org/10.1172/JCI204119DS1). At the time of tumor resection, all but 3 of the NETs in our cohort were treatment naive (Figure 1B). Among the 3 with prior therapy exposure, 2 patients had received somatostatin analog therapy, and 1 PanNET patient had received combination capecitabine/temozolomide therapy. Of all the samples in the cohort, 1 PanNET was classified as WHO grade 3 (G3); the remaining tumors were classified as WHO G1 or G2 (Figure 1B).

Next, we defined the cellular landscapes of PanNETs and siNETs captured within our multiomic snRNA-seq data. After quality control and preprocessing, our snRNA-seq dataset included 217,636 high-quality nuclei from 36 of our 38 samples (Figure 1C; see Methods). Leiden clustering and canonical marker gene expression were used to define broad cell types, including immune, endothelial, cancer-associated fibroblast, exocrine, and epithelial cells (Figure 1C and Supplemental Figure 1A). Then, we combined inferred copy number alterations with expression of established NET markers to distinguish malignant NET cells from nonmalignant populations (see Methods; Supplemental Figure 1B). We also obtained 74,004 single-nuclei chromatin accessibility profiles from 33 of our 38 samples (Figure 1C) after quality control and filtering. Broad cell types were assigned using label transfer from matched snRNA-seq barcodes and clustering (see Methods). Malignant cells composed the largest proportion of cell types recovered in both snRNA-seq and snATAC-seq data (Figure 1C). Among microenvironmental populations, cancer-associated fibroblasts were the most frequently recovered cell types, followed by normal exocrine cells, endothelial cell types, and immune cell populations (Figure 1C).

### GEP-NET malignant cells comprise a spectrum of neuroendocrine transcriptional phenotypes.

Well-differentiated GEP-NETs are currently classified based on anatomical site and histologic grade. However, this classification does not account for tumor-intrinsic transcriptional heterogeneity nor does it reflect potential differences in cell states that may influence behavior or therapeutic response. Genomic studies have identified recurrent alterations in *MEN1*, *ATRX*, *DAXX*, and mTOR pathway genes in PanNETs (9–11), while siNETs typically harbor few recurrent somatic driver mutations (12). Yet, the extent to which transcriptional and epigenetic programs shape conserved and divergent tumor phenotypes across NETs remains unresolved.

To gain understanding of the transcriptional and epigenetic programs within NET cells, we isolated the malignant cells and applied consensus non-negative matrix factorization (cNMF) separately to siNET and PanNET malignant cells to uncover recurrent transcriptional programs (Figure 2, A and B; see Methods). In siNETs (*n* = 76,010 tumor cells from 16 tumors), we identified 3 robust cNMF programs (Figure 2A and Supplemental Table 2). The first program (si-cNMF1) was enriched for neuronal and synaptic signaling genes, including *KCNJ3*, *RBFOX1*, *DLG2*, *NRXN1*, and *CADPS*, as well as calcium channel subunits (*CACNA2D1*) and neurodevelopmental transcription factors (TFs; *SOX5* and *DAB1*). Through GSEA, we identified that this program was enriched for pathways involved in apical junction, mitotic spindle, and L-2/STAT5 signaling (Figure 2C). The second program (si-cNMF2) reflected secretory neuroendocrine identity, marked by top associated genes *CHGA*, *CHGB*, *PCSK1*, *SCG5*, and *TAC1*, and was enriched for oxidative phosphorylation, adipogenesis, and mTORC1 signaling pathways. The third program (si-cNMF3) displayed features of epithelial-to-mesenchymal transition and tumor progression, with top associated genes *ZEB2*, *ROBO1*, *DOCK4*, and *AHNAK*, along with stem-like (*TCF4*) and stress-responsive genes (*LGALS3* and *NEAT1*).

Similarly, we identified 4 cNMF programs in PanNET cells (*n* = 100,809 tumor cells from 20 tumors; Figure 2B and Supplemental Table 3). These included a neuronal-like program (p-cNMF1) with top associated genes *RIMS2*, *NRXN1*, *MEIS2*, *FGF14*, and *NBEA* and TFs *NPAS3*, *TFAP4*, and *ISL1*. This program was enriched for protein secretion, hedgehog signaling, and apical junction pathways (Figure 2D). The secretory neuroendocrine program (p-cNMF2) mirrored that seen in siNETs, with expression of *CHGA*, *CHGB*, *SCG2–5*, *TTR*, and *CPE* as well as enrichment for oxidative phosphorylation, MYC targets, and mTORC1 signaling pathways. A third program (p-cNMF3) corresponded to a proliferative state with top associated genes *MKI67*, *CDK1*, *CCNB1*, *AURKA*, *BIRC5*, *CDC20*, and *TOP2A* and was enriched for G_2_/M checkpoint and E2F targets. The final program (p-cNMF4) represented an epithelial-to-mesenchymal transition and stromal state defined by *VIM*, *FN1*, *COL1A1*, *COL3A1*, *SPARC*, and other matrix remodeling genes.

To determine whether these cNMF programs reflected shared cellular states across NET types, we also performed integrated cNMF on pooled siNET and PanNET cells. This analysis recapitulated the major cNMF programs identified independently as outlined above and demonstrated that each program included cells from both tumor types (Supplemental Figure 2). 2D density plots of normalized program scores (neuronal-like neuronal-like vs. secretory neuroendocrine secretory neuroendocrine) demonstrated that respective malignant siNET or PanNET cells form a continuous distribution across the 2 programs (cNMF1/2) rather than resolving into distinct subtypes (Figure 2, E and F). These findings support a model in which, regardless of the organ site, GEP-NET malignant cells span a conserved transcriptional spectrum anchored by neuronal-like and secretory neuroendocrine identities.

### Divergent epigenetic landscapes drive organ site–specific tumor programs.

We next asked whether this conserved transcriptional continuum among the 2 predominant programs was regulated by shared or organ site–specific epigenetic programs. We leveraged matched ATAC-seq data and performed TF binding motif enrichment analysis on accessible chromatin regions associated with each cNMF program, stratified by originating organ type (see Methods). Despite transcriptional convergence with respect to the cNMF program enrichments, siNETs and PanNETs displayed distinct epigenetic regulatory signatures. In siNETs, malignant cells most enriched for the neuronal-like cNMF program (si-cNMF1) had accessibility around binding motifs for TFs *MAX:MYC* and *MITF* as well as circadian regulators such as *CLOCK* and *TP63*, suggesting a stress-responsive, neurodevelopmental regulatory network similar to sensory or peripheral neurons (Figure 3A) (13, 14). The secretory program (si-cNMF2) was enriched around binding motifs for TFs *HOXA9*, *JUN:FOS*, and *SOX9*, which are linked to lineage plasticity, stem-like states, and epithelial remodeling (Figure 3B) (15, 16). When examining the effect on gene expression, we observed that the enriched TF binding motifs were associated with increased expression of their predicted target genes (Figure 3C). This observation reinforces the link between regulatory motif accessibility and downstream transcriptional expression, illustrating how distinct TF networks shape the neuronal-like versus secretory cNMF programs.

By contrast, in PanNETs, the malignant cells most enriched for the p-cNMF1 program displayed chromatin accessibility around binding motifs for TFs *TFAP4*, *ISL1*, and *NR3C2*, which are involved in foregut and pancreatic development (Figure 3D) (17, 18). PanNET malignant cells most enriched for the secretory neuroendocrine program (p-cNMF2) displayed accessibility around hindbrain- and midbrain-associated TF binding motifs, including *MSX2*, *LBX2*, and *EN2*, suggesting an alternate developmental origin for regulatory control (Figure 3E) (19–21). Importantly, these TF binding motif enrichments were largely nonoverlapping between siNETs and PanNETs despite the similarity in associated cNMF programs, indicating that the convergent transcriptional programs arise within divergent epigenetic frameworks shaped by tissue of origin. Within our snRNA-seq data, we observed that enrichment of TF regulons from a given malignant program corresponded to differential expression of its predicted gene targets in cells scoring highly for that same program (Figure 3F).

These findings suggest that while siNETs and PanNETs converge on a common axis of transcriptional malignant cell programs, spanning neuronal-like to secretory neuroendocrine, the upstream epigenetic regulatory circuits associated with these malignant cell programs are organ specific. Notably, the secretory neuroendocrine program in both tumor types, which is defined by classic neuroendocrine identity, was also enriched for regulatory motifs associated with developmental plasticity and migration, raising the possibility that this transcriptional state may be preferentially selected or stabilized within organ-specific epigenetic contexts during tumor progression.

### Spatial coupling of malignant programs with distinct microenvironmental niches.

To further understand how these GEP-NET transcriptional programs are shaped by and influence the tumor microenvironment, we next assessed the spatial organization of the usage of these transcriptional programs using 10x Visium spatial transcriptomics. For these samples, we categorized spatial transcriptomic spots as pure tumor regions, mixed regions containing both malignant cells and tumor microenvironment cells in similar proportions, or regions of normal tissue using copy number alteration profiles inferred from the spatial data (see Methods).

Consistent with pathologist annotation and similar to the single-nucleus multiome cell data, the spatial transcriptomic data had a high prevalence of malignant cells (Figure 4A). We next performed gene signature scoring for the 3 siNET and 4 PanNET malignant cell cNMF programs on the inferred tumor spots and found that they displayed distinct spatial distributions within the GEP-NET samples (see Methods). To further characterize spatial organization, we conducted distance-based analyses to evaluate the localization of malignant spots enriched for each cNMF program relative to the tumor boundary, as well as their proximity to neighboring cell types inferred from adjacent spots (see Methods). Notably, malignant spots with high cNMF2 expression were preferentially localized deeper within the tumor interior, whereas cNMF1-high malignant spots were more frequently found closer to the tumor periphery (Figure 4B), indicating that conserved malignant transcriptional programs occupy distinct spatial niches within GEP-NETs.

When examining the histology of the areas enriched for the cNMF programs, we found that malignant spots scoring highest for si-cNMF1 were specifically localized in areas with high density of tumor cells, while malignant spots scoring highest for si-cNMF2 were present in areas with stromal infiltration (Figure 4, C and D, and Supplemental Figure 3, A–C). For PanNET samples, we likewise observed spatial localization of the malignant spots scoring highest for the respective cNMF programs, where malignant spots scoring most highly for p-cNMF1 were positioned in areas with high tumor cell density and malignant spots scoring most highly for p-cNMF2 comprised an area of cells with degraded cytoplasms (Figure 4, E and F). Transcriptional profiles of the spots confirmed these spatial patterns. Malignant spots scoring highest for the secretory neuroendocrine program showed significantly increased expression of genes involved in extracellular matrix remodeling, including *COL1A1*, *SPARC*, and *MMP2* (FDR-adjusted *P* = 0.015, paired Wilcoxon’s test across samples), supporting localization within stroma-remodeling niches. In contrast, neuronal-like–high malignant spots localized to densely cellular tumor regions with relative depletion of stromal infiltration. Although Visium spot resolution precludes definitive cell–cell contact inference, these malignant programs were derived independently from single-nucleus transcriptomic data prior to spatial projection, supporting that these patterns reflect biologically meaningful regional organization of malignant transcriptional states rather than de novo clustering driven by spot-level admixture alone. Together, these findings suggest that conserved malignant transcriptional programs occupy distinct fibrovascular and tumor-dense spatial microenvironments within GEP-NETs.

### Secretory neuroendocrine malignant programs are enriched for vascular interaction, stromal remodeling, and metastatic dissemination.

GEP-NETs have uniquely high vascularization among solid tumors, a feature that underpins their pathophysiology and positive clinical responses to antiangiogenic therapies such as sunitinib, bevacizumab, surufatinib, and cabozantinib (13, 22–25). However, the NET phenotypes that drive vascular remodeling and their spatial coordination with stromal compartments remain incompletely defined.

To investigate how the identified malignant cNMF programs may influence how the malignant cells coordinate with other microenvironmental cell types to support NET invasion and progression, we performed ligand-receptor interaction analysis within the snRNA-seq data (see Methods). Across both siNET and PanNET datasets, secretory neuroendocrine–high malignant cells exhibited enriched paracrine signaling interactions with endothelial cells and fibroblasts, particularly through pathways known to mediate angiogenesis, fibrosis, and extracellular matrix remodeling (Supplemental Figure 4). Top-ranked interactions included *TGFB1-ITGB1*, *VEGFA-FLT1*, and *LAMA2-ITGA1*, suggesting secretory neuroendocrine–high malignant cells may be engaged in the activation of perivascular and stromal niches (26–28).

We next explored how these interactions might relate to the spatial context with our spatial transcriptomic data. Across all samples, secretory neuroendocrine–high malignant spots demonstrated increased spatial association with endothelial and stromal compartments relative to neuronal-like–high regions (*P* < 0.05; Wilcoxon’s 2-sided test) (Figure 5A). For example, in a representative PanNET sample (P17), endothelial cells were consistently localized next to cNMF2-high (secretory) malignant spots (Figure 5, B and C). These regions exhibited elevated expression of profibrotic (*TGFB1* and *ANGPT1*), matrix-remodeling (*MMP2* and *COL1A1*), and angiogenic genes (*VEGFA* and *SPARC*) (*P* = 8.23 × 10^–3^; Wilcoxon’s rank-sum test) (Figure 5D). Moreover, endothelial cells in these regions expressed high levels of *FLT1*, *TEK*, and *TGFBR2*, supporting active paracrine signaling with adjacent tumor cells. Relatedly, the ligand-receptor interaction analysis within the snRNA-seq data predicted tumor-endothelial communication, with conserved ligand-receptor pairs that promote neovascularization and vascular permeability (Supplemental Figure 4) (29). Notably, *TGFB1* expression was polarized, with high expression in secretory neuroendocrine–high malignant spots but with negligible expression in neuronal-like–high malignant spots, paralleling *ITGB1* expression patterns (Figure 5, E and F). In addition, TF binding motif enrichment analysis of the snATAC-seq data revealed increased accessibility at *AP-1* (*JUN:FOS*) and *SMAD* motifs in secretory neuroendocrine–high malignant cells, TFs that act as downstream effectors of cellular stress and TGF-β signaling pathways. Given the established roles of *AP-1* and TGF-β signaling in extracellular matrix remodeling and stromal tumor interactions (26, 30), these findings are consistent with a chromatin landscape that may be permissive to stromal engagement in cNMF2-high malignant cells, rather than indicative of active regulatory control.

To further address the spatial resolution limitations of Visium, we generated Xenium in situ spatial transcriptomic data from 2 siNET (S4 and S9) and 2 PanNET (P17 and P18) specimens from patients in our cohort to provide orthogonal single-cell-resolution cNMF2 spatial context (Supplemental Methods). Because the Xenium panel was targeted and lacked several canonical neuroendocrine and cNMF-associated genes, Xenium data were not used to independently reconstruct the malignant transcriptional programs. Instead, we calculated representative Xenium marker scores using overlapping genes between the Xenium panel and the single-nucleus–derived cNMF programs and used these data conservatively to assess the stromal architecture underlying Visium-defined program-enriched regions.

Across multiple samples, malignant cells with high cNMF2-associated Xenium marker scores localized within stroma-rich tumor regions and demonstrated increased proximity to endothelial and fibroblast populations, particularly in tumors with extensive stromal infiltration and fibrotic architecture (Supplemental Figures 5 and 6). High-resolution Xenium spatial mapping further resolved the fibrovascular organization underlying cNMF2-enriched regions, revealing endothelial, fibroblast, and myeloid populations immediately adjacent to malignant tumor cells in areas corresponding to Visium-defined secretory neuroendocrine–high regions (Supplemental Figure 7). Together, these findings support that the stromal and vascular associations identified by Visium reflect biologically meaningful regional microenvironmental organization rather than solely spot-level cellular admixture.

Together, these findings position the secretory neuroendocrine malignant cell program as a malignant state associated with stromal and vascular compartments in GEP-NETs that may contribute to regional extracellular matrix remodeling and vascular co-option. The spatial and molecular features of the secretory neuroendocrine program may help explain the clinical efficacy of antiangiogenic therapies in GEP-NETs and further highlight the tumor–endothelium interface as a potentially actionable therapeutic niche.

### Neuronal-like malignant programs define stroma-sparse tumor states with immune cell–associated features.

In contrast to the stroma-interactive phenotype of the secretory neuroendocrine malignant cell program, neuronal-like–high malignant cells had predicted interactions with myeloid populations, particularly macrophages and monocytes. The described ligand-receptor interaction analysis revealed conserved neuroimmune signaling across neuronal-like–high malignant cells, including *CX3CL1-CX3CR1*, *IL34-CSF1R*, and *CSF1-CSF1R*, pathways known to regulate microglial-neuronal crosstalk, monocyte recruitment, and innate immune niche formation (Supplemental Figure 4) (31–34). Additionally, neuronal-like–high malignant cells expressed neuronal ligands such as *NRXN1*, *DLG2*, and *RIMS2*, highlighting enrichment of neuronal communication–related genes within immune cell–interactive malignant regions (Supplemental Figure 8) (35, 36).

The spatial transcriptomic data supported this neuroimmune signaling–associated state. Malignant spots scoring highest for the neuronal-like program preferentially localized to densely cellular tumor regions that were relatively depleted of stromal and fibrovascular infiltration in both siNETs (Figure 6A) and PanNETs (Figure 6B). In a representative siNET (sample S4), si-cNMF1–high malignant spots (neuronal) were positioned within an immune cell–dense cluster (Figure 6, C and E). The malignant spots in these immune cell–enriched regions lacked enrichment of si-cNMF2, and these regions were notably devoid of fibrotic or vascular features, reinforcing a distinct immune cell–permissive microenvironment. By contrast, PanNETs, which are characteristically more vascularized, exhibited higher immune infiltration overall, yet still displayed a reciprocal pattern where p-cNMF2–high malignant spots were present in regions of low immune density, as observed in sample P21 (Figure 6, D and F).

Orthogonal Xenium analyses similarly supported the tumor-dense architecture of neuronal-like–enriched regions. Across samples, malignant cells with high cNMF1-associated Xenium marker scores localized to comparatively stroma-sparse regions characterized by high tumor cellularity and relative depletion of fibrovascular organization (Supplemental Figures 5–7). Although immune proximity varied between tumors, subsets of cNMF1-enriched regions demonstrated spatial association with nearby myeloid populations, consistent with the neuroimmune ligand-receptor interactions predicted by the single-nucleus analyses. These findings further refine the regional architecture inferred from Visium and suggest that neuronal-like malignant states occupy a distinct tumor-dense microenvironment with heterogeneous immune engagement that warrants further mechanistic investigation.

Collectively, these findings suggest that neuronal-like malignant cells occupy a spatially distinct, tumor-dense microenvironment enriched for neuronal signaling and neuroimmune interaction pathways, while remaining comparatively depleted of the fibrovascular architecture associated with the secretory neuroendocrine program. Although localized immune interactions were observed in subsets of tumors, stromal and endothelial enrichment surrounding secretory neuroendocrine–high malignant regions represented the most reproducible spatial phenotype across datasets. These divergent malignant cell ecosystems raise the possibility that effective treatment of GEP-NETs may ultimately require therapeutic strategies tailored to both the malignant transcriptional state and its associated microenvironmental context.

### Malignant tumor programs associate with NET clinical stage and disease progression.

Lastly, we evaluated how the malignant cNMF programs relate to clinical stage and tumor progression in both siNETs and PanNETs. Across both tumor types, we observed a consistent pattern of higher neuronal-like program scores in localized primary tumors and higher secretory neuroendocrine program scores in metastatic lesions, although these differences did not reach statistical significance after correction for multiple comparisons (Figure 7, A and B).

In siNETs, this shift was most pronounced along the metastatic continuum, with si-cNMF2 scores increasing from regional to distant metastatic disease. Consistent with this observation, malignant cNMF program scores were also associated with metastatic status at diagnosis, with higher secretory neuroendocrine and lower neuronal-like scores observed in patients presenting with metastatic disease (Supplemental Figure 9). These patterns were reproducible within individual tumors sampled across progression states, including a representative siNET sample (S8) that exhibited a stepwise increase in si-cNMF2 and concomitant decrease in si-cNMF1 from primary to regional and distant metastatic lesions (Supplemental Figure 9).

To further validate the association between malignant cell programs and tumor progression, we analyzed an independent external bulk RNA-seq dataset comprising 22 localized and metastatic primary PanNET samples (37). In this independent dataset, metastatic tumors exhibited significantly lower neuronal-like program scores compared with primary tumors (Holm-adjusted *P* = 0.026; Figure 7C). Secretory neuroendocrine program scores were also higher in metastatic tumors, consistent with the direction observed in our discovery cohort, although this difference did not reach statistical significance after correction for multiple comparisons. Together, these findings demonstrate that these malignant cNMF programs exhibit distinct clinical associations with primary and metastatic NETs.

## Discussion

Here, we present the largest and most comprehensive single-nucleus and spatial transcriptional profiling study of malignant cells in GEP-NETs to date. We integrated single-nucleus multiomic and spatial transcriptomic data to reveal the transcriptional, epigenetic, and spatial features that shape NET heterogeneity. To our knowledge, this study is the first to place malignant cell states in their spatial context across GEP-NETs, allowing us to associate the identified malignant cell programs and the niche-specific tumor microenvironment interactions predicted that drive NET growth and progression.

Altogether, our analyses identified 2 conserved malignant cell cNMF programs that structure heterogeneity across PanNETs and siNETs. Importantly, although these programs were conserved across organ sites, their relative abundance, spatial organization, and associated microenvironmental interactions varied substantially between tumors and patients, highlighting that GEP-NET heterogeneity is shaped not only by distinct genomic backgrounds but also by differences in malignant cell state composition and regional niche context. Despite their distinct genomic landscapes, as described in prior studies (6), we found that tumors from both organ sites converge on neuronal-like (si-cNMF1/p-cNMF1) and secretory neuroendocrine (si-cNMF2/p-cNMF2) programs, underscoring a shared architecture of NET biology. We further discovered that these programs are active, functional states characterized by distinct epigenetic regulation, spatial localization, and clinical associations.

The secretory neuroendocrine program was enriched within fibrovascular stromal niches, where it engaged extracellular matrix remodeling, angiogenic, and stroma-interactive pathways. Its spatial localization and enrichment in metastatic tumors suggest that the secretory neuroendocrine program represents a metastasis-enabling state, potentially driven by TGFB–integrin crosstalk and other stromal interactions. In contrast, the neuronal-like program localized to high-cell-density, immune cell–interactive regions and associated with calcium signaling and neuronal TF motifs. This program was enriched in nonmetastatic tumors, consistent with a more plastic, progenitor-like phenotype capable of immune engagement. Together, these findings support a model in which GEP-NET progression is associated with a shift in the relative prevalence of transcriptional programs, with advanced and metastatic tumors preferentially enriched for the secretory neuroendocrine program. The neuronal-like program, with lineage-restricted TF (e.g., *TFAP4*, *MITF*, *Isl1*) binding motif enrichment, may represent a more differentiated, tissue-committed program. By contrast, secretory neuroendocrine, marked by *HOX*, *AP-1*, and developmental TF binding motif enrichment, displays hallmarks of dedifferentiation and plasticity, enabling cells to remodel their identity and adapt to stress. This dichotomy mirrors paradigms in other solid tumors, where conserved and transient transcriptional states provide a more powerful framework for understanding disease progression than mutational profiles alone (38). Importantly, this neuronal-like–to–secretory neuroendocrine continuum should be interpreted as a spectrum of malignant cell transcriptional states rather than definitive evidence of distinct developmental lineages or cellular origins. While these findings raise the possibility of developmental plasticity or lineage specialization within GEP-NETs, the current study was not designed to directly resolve lineage relationships.

More broadly, our findings suggest that GEP-NET heterogeneity may be best understood through a cell state framework in which conserved transcriptional programs coexist in varying proportions across tumors rather than through discrete static subtypes alone. While prior classification systems have emphasized tumor origin, mutational status, or proliferation index, our analyses demonstrate substantial variability in the relative enrichment, spatial distribution, and microenvironmental coupling of these conserved programs both within and across patients. Thus, the neuronal-like and secretory neuroendocrine programs identified here may represent recurring endotype building blocks that collectively generate the broader phenotypic heterogeneity observed across GEP-NETs. The coexistence of these states within individual tumors further raises the possibility that malignant cells dynamically transition between programs in response to local stromal, immune, or vascular cues during tumor progression.

The spatial localization of the secretory neuroendocrine program within stromal and vascular niches further raises the possibility that regional microenvironmental properties contribute to malignant cell state regulation in GEP-NETs. Recent studies in mechanogenomics have demonstrated that extracellular matrix organization, tissue stiffness, and integrin-mediated signaling can shape transcriptional plasticity and metastatic competence across solid tumors (39). In this context, the enrichment of extracellular matrix remodeling pathways, TGFB signaling, laminin-integrin interactions, and proangiogenic programs within secretory neuroendocrine regions suggests that these stromal niches may provide both biochemical and biomechanical cues that reinforce this malignant cell state. Conversely, the neuronal-like program localized to densely cellular, relatively stroma-sparse regions with evidence of variable immune engagement, supporting a model in which distinct spatial microenvironments contribute to the emergence and maintenance of divergent malignant cell phenotypes within individual tumors.

Orthogonal single-cell-resolution Xenium spatial profiling further supported the regional niche architecture inferred from the Visium analyses. Although the targeted Xenium panel did not permit independent reconstruction of the full cNMF programs, these data supported the spatial organization of program-enriched regions by demonstrating stroma- and endothelium-rich microenvironments surrounding secretory neuroendocrine–associated malignant cells, while neuronal-like–associated malignant regions localized to comparatively tumor-dense and stroma-sparse areas with heterogeneous immune proximity. Together, these complementary spatial approaches support the presence of biologically organized malignant cell ecosystems and suggest that the spatial associations observed in Visium reflect regional microenvironmental organization rather than solely technical limitations related to spot size. Although additional functional studies and expanded cohort profiling studies are necessary to directly interrogate these relationships in GEP-NETs, our findings further support the concept that tumor progression is shaped not only by intrinsic transcriptional programs, but also by the regional microenvironmental contexts in which these programs reside.

We also identified a small proportion of tumor cells in our cohort that express a proliferative program encompassing cell cycle genes such as *MKI67*, *TOP2A*, *CDK1*, and *AURKA*, among others. Although GEP-NETs are slow-growing neoplasms, proliferating tumor cells are present within these tumors that contribute to their overall growth and metastasis; the percentage of Ki67^+^ cells within a GEP-NET defines its WHO grade. By including this proliferative population, our cohort captures the full range of transcriptional diversity across GEP-NET malignant cells.

Our results also extend prior NET classifications based on genotype or proliferation index by highlighting a unifying cell state model that transcends the originating organ site. Importantly, we identify putatively actionable signaling pathways within these states, including TGFB–integrin crosstalk in the secretory neuroendocrine program and myeloid-tumor interactions in the neuronal-like program. Therapeutically, such features open potential opportunities for intervention. These include myeloid-directed therapeutics currently in clinical development that could be used to disrupt tumor-myeloid interactions in neuronal-like NETs. The spatial confinement of these niches may also enable localized delivery of immunomodulatory agents while sparing stromal and vascular compartments. These insights open avenues for therapeutic strategies that target tumor-microenvironment interactions or exploit plasticity in NET differentiation states.

Limitations to this study include the relatively modest number of spatial transcriptomic samples and the need for longitudinal data to fully define the dynamics of program switching during progression or therapy. We were also limited in the spatial resolution of the data in this study. Although we incorporated Xenium profiling in a subset of samples to provide orthogonal single-cell-resolution spatial context, broader application of emerging higher-resolution spatial technologies that enable single-cell-resolution full transcriptome analysis across larger cohorts will be important for more comprehensively resolving rare cellular populations, fine-scale tumor microenvironment interactions, and dynamic spatial transitions between malignant cell states (40).

An additional important limitation is the lack of robust and widely validated in vitro and in vivo preclinical models for GEP-NETs, which currently constrains mechanistic and functional interrogation of the pathway-specific and microenvironment-linked vulnerabilities identified in this study. Although recent advances have led to the development of emerging NET model systems, including patient-derived and organoid-based approaches, further validation is necessary to determine how faithfully these models recapitulate the molecular, transcriptional, and microenvironmental features of human gastrointestinal NETs (41). Future studies leveraging these evolving model systems are critical for functionally testing the therapeutic dependencies and stromal or immune interactions nominated by our spatial multiomic analyses.

Finally, although we identified conserved malignant cell programs across GEP-NETs, the full extent of biological heterogeneity present across patients, metastatic sites, and treatment states is likely broader than captured in our cohort. Rare malignant subpopulations, patient-specific states, and transient intermediate programs may have been underrepresented due to cohort size, sampling bias, and technical limitations inherent to the snRNA-seq and Visium approaches. Future studies incorporating larger longitudinal cohorts and higher-resolution spatial technologies are important for resolving the full spectrum of GEP-NET heterogeneity and malignant cell state plasticity.

In summary, our study establishes a cross-tissue framework for understanding NET biology through conserved malignant cell programs. By linking these programs to spatial niches, microenvironmental interactions, and clinical stage, we provide a roadmap for tumor progression and therapeutic targeting in GEP-NETs.

## Methods

### Sex as a biological variable

This study included tumor specimens from both male and female patients, and patient sex was recorded as part of the clinical characteristics of the cohort. Similar findings are reported for both sexes.

### Experimental methods

#### Sample collection and nuclei isolation for snRNA-seq and snATAC-seq.

Fresh tumor resection tissue was collected from patients who provided written informed consent at Brigham and Women’s Hospital. Multiple pathologists in the Department of Surgical Pathology at Brigham and Women’s Hospital identified tumor tissue within each resection, which was dissected away from normal tissue using a scalpel. Tumor tissue that was not needed for clinical pathology review was snap-frozen and held in liquid nitrogen tanks for long-term storage.

To isolate nuclei for joint multiomic snRNA-seq/snATAC-seq, a portion of each snap-frozen tumor sample was cut off with a scalpel and placed into 1.5 mL Eppendorf tubes on dry ice. 1 mL of TST buffer was then added to the tube. Tumor samples were minced in the tube into < 0.5 mm fragments with spring scissors for 7 minutes on ice, followed by 3 minutes of mechanical dissociation by pipetting the tumor buffer mixture up and down using a P1000 tip. At the end of the 10-minute mincing period, the contents of the tube were transferred to a 30 μm filter (MACS SmartStrainers, Miltenyi, 130-098-458) attached to a 15 mL Falcon tube on ice. An additional 1 mL of TST was used to rinse the Eppendorf tube and the 30 μm filter. The filter was then rinsed with 3 mL of 1× TST buffer, bringing the total suspension volume to 5 mL.

Next, the suspension was centrifuged in a swinging bucket rotor for 5 minutes at 500*g* and 4°C with the centrifuge deceleration ramp set to 5 (out of maximum 9). The 15 mL tube containing the cell pellet was placed on ice, and the supernatant was carefully removed using P1000 and P200 pipettes. To lyse cells, 100 μL of 0.1× lysis buffer was added to the pellet, which was gently dissociated by pipetting 5 times with a P200 pipette. After 2 minutes, 1 mL of wash buffer was added and gently pipetted 5 times to prevent excessive lysis. The resulting suspension was centrifuged in a swinging bucket rotor for 5 minutes at 500*g* and 4°C with the centrifuge deceleration ramp set to 5.

The supernatant was again carefully removed and stored in a 1.5 mL Eppendorf tube. Depending on the size of the final pellet, 30–50 μL of 1× Diluted Nuclei Buffer (10x Genomics) was added to the pellet and gently mixed 5 times using a P200 pipette on ice to ensure minimal nuclei clumping. The nuclei suspension was further diluted 1:10 in Diluted Nuclei Buffer prior to quantification. Acridine orange/propidium iodide staining was performed, and 5 μL of the diluted nuclei suspension was loaded into a LUNA-FL Dual Fluorescence Cell Counter (Logos Biosystems) for nuclei counting and assessment of lysis completeness and debris.

#### Joint snRNA-seq/snATAC-seq.

After counting and quality assessment, nuclei from each sample were prepared for single-cell multiomic sequencing using the protocol outlined in the 10x Genomics Chromium Next GEM Single Cell Multiome ATAC + Gene Expression User Guide, Rev. A, targeting barcoding of 10,000 nuclei per sample. Transposition mix from the Chromium Next GEM Single Cell Multiome ATAC + Gene Expression Reagent Kit (10x Genomics, 1000283) was added to the nuclei stock, followed by 60 minutes of incubation in a thermal cycler at 37°C. Transposed nuclei were then combined with Master Mix and loaded into the Chromium Next GEM Chip J (10x Genomics, 2000264) along with Single Cell Multiome Gel Beads (10x Genomics, 2000261). The chip was run on the 10x Chromium controller using the standard Multiome program to (a) generate barcoded, full-length cDNA that is amplified for downstream snRNA-seq gene expression (GEX) library preparation and (b) barcode transposed DNA for downstream snATAC-seq library preparation.

For both tumor types (siNET and PanNET), GEX libraries from all samples were pooled in groups of up to 8 samples and sequenced according to the parameters outlined in the Single Cell Multiome User Guide. snATAC-seq libraries were separately pooled in groups of up to 8 samples and sequenced with appropriate parameters. All single-cell GEX and ATAC-seq libraries were sequenced on S1-100 and SP-100 flow cells using an Illumina NovaSeq 6000 sequencer.

#### Spatial transcriptomic sequencing.

H&E slides were prepared from FFPE blocks of all 37 tumors, which were then reviewed by a board-certified pathologist to select high-quality samples for spatial transcriptomic sequencing. Exclusion criteria for FFPE samples consisted of paucity or absence of tumor cells, high density of scars or fibrosis, and tissue degradation, tearing, or shredding.

To determine the quality of RNA recovery from our selected samples, we generated FFPE curls from each tumor block, which were placed in 1.5 mL Eppendorf tubes on ice. RNA extraction from each sample’s curls was performed according to the 10x Genomics Visium CytAssist RNA extraction protocol. Agilent Bioanalyzer traces of each sample’s extracted RNA were used to calculate DV200 scores for each sample.

Next, we used the 10x Genomics Visium Spatial Gene Expression protocol to perform spatial transcriptomic sequencing of NET samples whose DV200 scores surpassed 10x Genomics’ recommended thresholds. 10 μm sections were cut from each FFPE block and placed on Fisher SuperFrost slides according to 10x Genomics’ Visium CytAssist specifications. The 10x Genomics CytAssist platform was then used to transfer the sectioned tissue to a 6.5 × 6.5 mm capture area on 10x Visium Spatial Gene Expression Slides. Visium Spatial Gene Expression libraries and H&E-stained slides from each sample were generated according to the 10x Genomics protocol; the GEX libraries were then sequenced on an Illumina sequencer.

### Computational methods

#### snRNA-seq and snATAC-seq data preprocessing.

Raw base call (BCL) files generated from all single-cell GEX and ATAC-seq libraries were individually demultiplexed and underwent read alignment, barcode counting, peak calling, and cell counting using the standard Cell Ranger ARC analysis pipeline (version 2.0; 10x Genomics) with default parameters. The prebuilt human genome reference included in Cell Ranger ARC (GRCh38-2020-A_arc_v2.0.0) was used for read alignment. Cell Ranger ARC produced raw and filtered count matrices whose rows contained all gene and peak features and whose columns included either all barcodes with non-zero GEX or ATAC-seq signal (raw matrix) or only barcodes identified as cells (filtered matrix).

#### snRNA-seq: ambient RNA decontamination, multiplet removal, and cell filtering.

Ambient RNA decontamination and empty droplet removal were performed on all samples using the CellBender software package, which uses an unsupervised deep-generative model to parse cell-containing droplets from empty ones to remove technical artifacts from cell-free RNA (42). The raw count matrix and estimated cell count produced by the Cell Ranger ARC pipeline were inputted into the *remove-background* module of CellBender, and all samples were run using the following default parameters: “ambient” model, low count threshold of 10, learning rate of 0.000001, 150 learning epochs, and false-positive rate of 0.01. For samples whose expected cell counts did not match the CellBender-estimated number of droplets, or whose unique molecular identifier (UMI) curve lacked an empty droplet plateau after running CellBender with default parameters, the *--total-droplet-barcodes* parameter was increased to 50,000–75,000. All other samples were run with this parameter set equal to the expected number of cells.

To remove “multiplets,” or technical artifacts caused by unintentional droplet encapsulation of 2 or more cells, we performed doublet detection and removal on the CellBender-cleaned count matrix using the Scrublet package (43) (Python, version 0.2.3). Multiplets within the count matrix were identified and discarded using an expected doublet rate of 0.1–0.2 and manually selected doublet score thresholds. Next, cells with fewer than 200 genes or more than 5% mitochondrial genes were removed from the count matrix; genes expressed in fewer than 3 cells in the sample were also removed. The resulting ambient RNA-diminished, singlet-only, filtered count matrix was used for all further downstream quality control and analysis.

#### Dimensionality reduction, clustering, and preliminary cell type identification.

The count matrix for each sample was log-normalized and scaled using the default *log(x+1)* approach. We then performed linear dimensional reduction on the normalized count matrix using principal component analysis (PCA) on the top 3,000 highly variable genes across all cells. The first 50 PCA components were used for Leiden clustering (resolution = 0.2). To visualize these preliminary clusters in 2D space, UMAP was performed using the same 50 PCA components. Differential gene expression was performed on a per-cluster basis to identify marker genes and assign preliminary cell type identities for each cluster.

#### Across-sample merging, identification of malignant cells, and low-quality cell removal.

After preliminary clustering, we concatenated the raw count matrices and metadata for all samples. This merged object underwent filtering as above to remove cells with < 200 counts, genes with non-zero counts in fewer than 3 cells, and cells with > 5% mitochondrial reads. We performed log normalization and PCA on the top 3,000 highly variable genes and dimensional reduction as described above. Broad cell type labels were determined by differential gene expression (2-sided Wilcoxon’s rank-sum test, 1 cluster vs. cells from all other clusters) on the merged, nonintegrated full object.

After we annotated all cells for their corresponding “normal” cell types, we utilized inferCNVpy (version 0.4.5; Trinity CTAT Project) to identify the malignant cells in each sample (inferCNV of the Trinity CTAT Project). Given the variable proportion of immune cells in each sample, we first generated a uniform normal cell reference dataset by randomly sampling 10% from all immune cells available across the cohort. We used this common reference as the control dataset for all per-sample inferCNVpy runs, and it was run with all mesenchymal cells as the “case” dataset for each sample. inferCNVpy was run on the log-normalized gene expression matrix using a sliding gene window of 250 genes, a copy number variation (CNV) score log fold-change clip of 0.1, and a step of 1 (thus computing all possible windows). Then, Leiden clustering on the cells’ CNV profiles was conducted on a neighborhood graph computed with 20 neighbors and 20 top principal components (PCs) at a resolution of 1. Otherwise, default parameters were used. This approach clusters cells based on their inferred copy number alteration profile and computes a CNV score for each of the clusters generated. We defined malignant cells as those that do not cluster with immune cells (<25% of the cluster are immune cells) and have a CNV score higher than 1 standard deviation above the mean CNV score of the immune cells. Finally, we performed manual review for each sample to confirm the presence of aberrant copy number alterations in the cells annotated as malignant.

For each sample, cells for which the cNMF annotation did not match the clustering annotation were removed. A cohort-wide control set was generated by randomly sampling from all immune cells. Neuroendocrine cells were filtered as the case set for each sample. InferCNV was run across all genes (window = 250 genes, log fold-change clip = 1, step = 1). InferCNV clusters cells based on their CNV profile and computes CNV score for each cluster. To process InferCNV results, cutoff was defined as mean + 1 SD CNV score of the control immune cells to filter for cells with significant copy number alterations in each sample’s case set.

#### Cell subtype annotation and visualization.

After defining broad cell lineages (e.g., tumor, immune, endothelial), we isolated the raw data corresponding to each cell type cluster from the integrated object to create new objects for analysis. We then performed de novo dimensionality reduction and clustering using the top 3,000 genes and 30 PCs, and Harmony integration was performed on each cell type object to reduce patient-specific batch effects. To generate cell subtype annotations, differential gene expression was performed using a 2-sided Wilcoxon’s rank-sum test to compare expression profiles of 1 cluster versus cells from all other clusters.

#### Malignant cell program discovery through cNMF and characterization.

To dissect the malignant cell compartment, we employed cNMF (44) per individual patient sample and then aggregated the results as described below. cNMF was performed on a sample level rather than on the full cohort to avoid detecting patient-specific programs primarily driven by technical factors such as batch effects or CNV profiles. For each sample, cNMF was performed on the RNA count matrix of the 2,000 most highly variable genes, selecting the number of components (k) based on recommended criteria (i.e., inspecting the error and stability plot and picking the smallest k that minimized error while maximizing stability). Density threshold was set to 0.1 for each sample. cNMF programs expressed in too few cells or showing expression of tumor microenvironment–related genes, potentially indicating contamination, were manually removed. The cNMF gene expression programs generated per individual patient sample were characterized by a vector of weights per gene representing its contribution to the program. These programs were combined across all samples by calculating their pairwise cosine similarity after removing small (high score in < 10 of cells) or contaminated programs. Hierarchical clustering with an average linkage method was then applied to group similar programs into 3 clusters for siNETs and 4 clusters for PanNETs. A cNMF program was defined by the median weight of clustered gene expression programs, with the top 200 contributing genes used as a gene signature for the cNMF program. Cells from all patients were scored for the resulting cNMF gene signatures using the Scanpy scoring method, i.e., the average gene expression of signature genes subtracted from the average gene expression of control genes. GSEA (45) was run using the prerank function on the ranked list of genes associated with each program, using the hallmarks of cancer as a search database.

#### snATAC-seq: quality control and multiplet removal.

For each sample, we first filtered the snATAC-seq fragment file produced by CellRanger ARC to contain only barcodes identified by CellRanger as bona fide cells. Peak calling was performed using MACS3 (Python, version 3.0.0bl; https://macs3-project.github.io/MACS/docs/callpeak.html). The resulting peak-by-cell matrix was then used to construct a Signac (46) object (R, version 1.10.0) after filtering for (a) nuclei with a transposition event in at last 200 peaks and (b) peaks detected in at least 10 nuclei.

To identify and remove doublets from the peak matrix, we scored each nucleus using the scDblFinder method (R, version 1.14.0) (47). Nuclei labeled as “doublet” were removed from the Signac object, and the multiplet-cleaned peak matrix was used for further processing.

Next, we computed the following 5 quality control metrics for each cell: (a) fraction of fragments in peaks, (b) the ratio of reads in genomic blacklist regions from the ENCODE project, (c) nucleosome signal score, (d) transcription start site enrichment score, and (e) total number of fragments in peaks. Nuclei with fewer than 1,000 total fragments in peaks were removed from the peaks-by-cell matrix. Outlier thresholding for the remaining metrics was determined on a per-sample basis using the median ± median absolute deviation (MAD) to set upper and lower bounds.

#### snATAC-seq: dimensionality reduction, clustering, merging, and integration of sample-level datasets.

Dimensionality reduction of snATAC-seq data was performed using Signac (R, version 1.11.0) (46). For each sample, term frequency–inverse document frequency normalization was performed on all cells passing quality control thresholds. All features were used to run singular value decomposition on the normalized matrix. Latent semantic indexing dimensions 2–30 were used for UMAP and downstream clustering; the first latent semantic indexing dimension correlated strongly with sequencing depth and was not included. Shared nearest neighbor–based clustering and differential gene activity were performed to identify broad cell types within each sample. For samples containing more than 1 cell type, peaks were called on a cluster-level basis using MACS3; for samples containing only 1 cluster, bulk peaks were called using MACS3.

To merge snATAC-seq data from the sample-level datasets, we first created a common set of peaks using the GenomicRanges reduce function on the cluster- or bulk-level peaks from each sample. These peaks were quantified and used to create new Seurat objects for each dataset. Next, we merged all Seurat objects per the default Signac workflow. Gene activity was inferred for all nuclei in the merged dataset using the *GeneActivity* function from Signac. Cell type annotations from matched snRNA-seq nuclei and differential gene activity between snATAC-seq clusters were combined to determine broad cell type annotations.

To refine peak representation within the merged object, broad cell types were used to re-call peaks on the integrated object with MACS3. These refined peaks were then used to construct a new Signac object, and low-quality cells were again excluded using the MAD-based thresholds above (peak region fragments > 1,000). Broad cell types were again annotated using a combination of differential gene activity and cell type labels from matched snRNA-seq metadata. The final Signac object contained 74,004 nuclei from 35 samples (20 PanNETs and 15 siNETs).

#### Motif enrichment analysis.

TF motif enrichment analysis of snATAC-seq data was performed using Signac (46, 48). To associate chromatin accessibility with malignant transcriptional programs, high-confidence cNMF program labels derived from matched snRNA-seq nuclei were transferred to snATAC-seq nuclei by matching cell barcodes. Malignant nuclei classified as cNMF1-high or cNMF2-high were analyzed separately for siNETs and PanNETs. Differentially accessible peaks between program-defined nuclei were identified using the *FindAllMarkers* function in Signac with a 2-sided Wilcoxon’s rank-sum test, retaining positive peaks with a minimum detection fraction of 0.01 and log fold-change threshold of 0.1. The top 200 differentially accessible peaks for each program were used for motif enrichment analysis. Motif DNA sequence information from the JASPAR 2020 collection was added to the Signac object using the hg38 genome (49). Motif enrichment was calculated using the *FindMotifs* function in Signac.

#### Spatial transcriptomic sequencing data preprocessing.

Processing of Illumina sequencing output of all CytAssist Spatial Gene Expression for FFPE libraries was performed using the 10x Genomics Space Ranger workflow. Raw Illumina BCL files were demultiplexed and converted to FASTQ files using the *spaceranger mkfastq* pipeline (version 2.1.0). Next, the *spaceranger count* pipeline was used to perform fiducial image alignment, tissue detection, read alignment (Space Ranger reference genome: GRCh38-2020-A), and barcode and UMI counting. Manual registration of the CytAssist and H&E image files was performed using the 10x Genomics Loupe platform. The filtered count matrix produced by Space Ranger was used for downstream quality control and analysis.

#### Spatial transcriptomics quality control, dimensionality reduction, and clustering.

Analysis of spatial transcriptomic data was performed using Squidpy (Python, version 1.3.1) (50). Visium spots with fewer than 5,000 or greater than 35,000 counts or with greater than 20% mitochondrial reads were filtered out of the Space Ranger–filtered count matrix. The remaining spots were normalized using SCTransformPy (https://github.com/atarashansky/SCTransformPy; commit ID 58fbc85b7067b1fb9a02486825f781a200552d45), and the top 5,000 highly variable genes were used for downstream PCA, UMAP, and Leiden clustering analysis (number of PCs = 50). cNMF programs were scored using AUCell scoring on the cancer cell compartments of each of the spatial transcriptomic samples.

To assess the spatial localization of malignant programs, we calculated the distance of each tumor spot to the tumor boundary in the Visium data. Malignant spots were classified as cNMF1-high or cNMF2-high using sample-specific program score thresholds, particularly taking the top 10th percentile for each. Euclidean distances from each spot to the nearest tumor–normal boundary were computed from Visium spatial coordinates, generating a distance-to-boundary measure for every spot. To account for differences in tumor size across samples, distances were normalized by the maximum distance within each sample, yielding a core periphery index (0 = tumor edge; 1 = deep core). Tumor edge was defined as the interface between malignant and nonmalignant regions based on spot-level classification. For each spot, distance to the tumor edge was calculated as the minimum distance to the nearest boundary spot on the Visium grid, providing a direction-independent measure of spatial proximity.

To quantify how malignant programs spatially associate with specific microenvironmental compartments, we computed the distance from each cNMF1-high or cNMF2-high tumor spot to the nearest immune, endothelial, or fibroblast spot in the Visium arrays. Distances were converted into “ring units,” defined as the mean nearest-neighbor spacing within each sample. Spots were then classified as near a given cell type if they fell within preset ring thresholds (≤1 ring). For each sample and malignant program, we calculated the fraction of tumor spots falling within these proximity thresholds. Differences between cNMF1-high and cNMF2-high tumor spots were evaluated using paired Wilcoxon’s signed-rank tests applied to per-sample fractions. Proximity fractions were visualized using bar plots comparing program-specific associations across samples.

#### Cell–cell communication analysis.

Cell–cell communication was inferred using LIANA (v1.5.1) with the CellChat statistical framework and CellChatDB (29, 51). Normalized snRNA-seq data were grouped by pathology subtype, and ligand-receptor interactions were scored for communication probability and statistical significance. Significant interactions were visualized with LIANA’s dot plot to identify program-specific signaling patterns.

#### Link between malignant programs and clinical characteristics.

To explore whether the identified cNMF programs correlated with clinical characteristics in our cohort, we evaluated program scores in malignant cells from the scRNA-seq data for siNETs and PanNETs. For patient-level analyses, cNMF program scores were averaged across malignant cells within each tumor, such that each tumor represented an independent observation. Patient-level program scores were then evaluated in relation to clinical characteristics, including primary versus metastatic disease.

#### Link between malignant programs and clinical characteristics in external bulk datasets.

To validate our findings, we assessed the scores of these programs in an external bulk RNA-seq dataset and examined their association with various clinical characteristics. RNA-seq expression data and clinical characteristics from the study by Greenberg et al. were used, which profiled untreated primary and metastatic pancreatic neuroendocrine tumors and represented the most clinically comparable publicly available bulk RNA-seq cohort at the time of analysis (37). Raw RNA-seq counts were transformed into transcripts per million, and the cNMF programs were scored using single-sample GSEA (42). The resulting program scores were then correlated with tumor node metastasis staging and metastatic status.

### Statistics

Statistical analyses were performed using R and Python. All statistical tests were 2 sided, and a *P* value < 0.05 was considered statistically significant unless otherwise indicated. Sample sizes were determined by the availability of high-quality patient specimens. Comparisons between groups were performed using nonparametric tests unless otherwise noted. Multiple hypothesis testing was controlled using the Benjamini-Hochberg FDR procedure. Additional details are provided in the figure legends.

### Study approval

Human patient sample collection was approved by the Dana-Farber Cancer Institute IRB (protocol 02-314). Informed written consent was acquired for patient participation, which was obtained per protocol. At the time of their clinic appointments, patients were contacted by a member of the study staff and were asked if they were interested in participating. The study was explained in detail, and informed consent was signed if the individual ultimately wished to participate. Patients specifically consented to (a) the collection of tissue and blood, (b) the collection and linkage of clinical data to the tissue, (c) recontact of the patient for the purpose of recruitment into new clinical protocols or for the purpose of obtaining clinical follow-up, (d) a one-time additional blood draw or urine collection for study purposes if not feasible in the context of a scheduled blood draw, and (e) collection of periodic blood samples.

### Data availability

Raw snRNA-seq and snATAC-seq data are available in the Database of Genotypes and Phenotypes under accession number phs003141.v2. Processed spatial data are available in Zonedo (https://zenodo.org/records/22062599). Code used for computational analysis is available in GitHub (https://github.com/vanallenlab/NET2.0; commit ID 1204d3b069f4d827dbbf329d14e94915e7f150be). Values for all data points in graphs are reported in the Supporting Data Values file. Further information and other requests should be directed to the corresponding author.

## Manuscript information

### Author contributions.

JK contributed to conceptualization, investigation, software analysis, formal analysis, visualization, and writing of the original draft. SEH contributed to conceptualization, data curation, investigation, software analysis, formal analysis, writing of the original draft, and review and editing of the manuscript. JK and SEH contributed equally to this work and share first authorship. Authorship order was determined based on the relative scope of contributions to the project. AG, DG, HIH, BMT, YT, EP, and TP contributed to investigation and review and editing of the manuscript. LV, KB, and RG contributed to investigation. LB and JLH contributed resources. ES contributed to project administration. JP contributed to conceptualization, investigation, and review and editing of the manuscript. JC provided resources and contributed to conceptualization, funding acquisition, data curation, supervision, and review and editing of the manuscript. EMVA provided resources and contributed to conceptualization, funding acquisition, supervision, writing of the original draft, and review and editing of the manuscript. JC and EMVA contributed equally to this work.

### AI disclosure.

The authors declare that no AI tools were used in the original research for or writing of this article.

### Conflict of interest.

EMVA serves on the Editorial Board for *Science Advances* and reports advisory and consulting relationships with Novartis Institute for Biomedical Research, Serinus Bio, TracerBio, Cellyrix; research support from Novartis and Bristol Myers Squibb; equity in Tango Therapeutics, Enara Bio, Manifold Bio, Microsoft, Monte Rosa, Riva Therapeutics, Serinus Bio, TracerBio, and Cellyrix; institutional patents (numbers US12404557B2, US20170115291A1, US11377696B2, WO2018183544A1, WO2019200329A1, WO2026009149A1, and WO2015013191A1) filed on chromatin mutations and immunotherapy response, and methods for clinical interpretation; and intermittent legal consulting on patents for Foley Hoag. JC reports advisory and consulting relationships with Boehringer Ingelheim, Curium, Lantheus, and Sanofi; research support from Camurus, Exelixis, and Lilly; equity in Merck; and honoraria from Ipsen and Sanofi. JLH is a consultant to Adaptimmune and Angiex.

### Funding.

This study was supported by the following:

- The Orr Family Foundation (to JK, SEH, JC, and EMVA).
- NIH 5U2CCA233195-05 (to EMVA).
- NIH R37 CA222574 (to JP and EMVA).
- NIH R01 CA227388 (to JP and EMVA).
- The Goldhirsh-Yellin Foundation (to JC).
- National Institute of General Medical Sciences award T32GM144273 (to SEH).

This work is the result of NIH funding, in whole or in part, and is subject to the NIH Public Access Policy. Through acceptance of this federal funding, the NIH has been given a right to make the work publicly available in PubMed Central.

## Supplementary Material

Supplemental data

Supplemental tables 1-3

Supporting data values

## Figures and Tables

**Figure 1:**
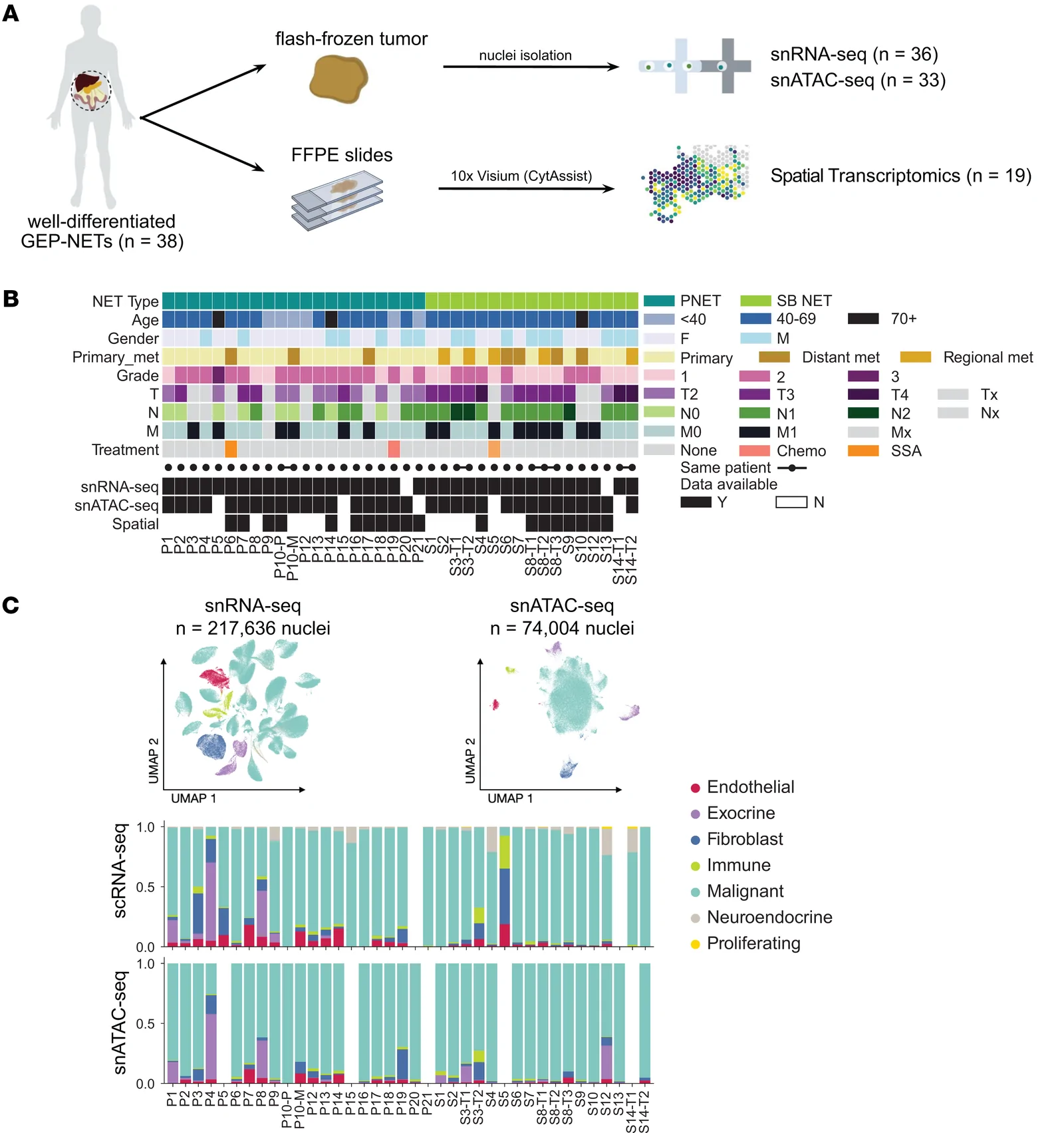
Multiomic characterization of well-differentiated PanNETs siNETs. (**A**) Multiome sample processing and sequencing workflow. Archival, flash-frozen NET samples were apportioned for nuclei isolation and subsequent joint snRNA-seq and snATAC-seq using the 10x Genomics protocol. FFPE slides were generated from each sample and used to generate spatial transcriptomic data using the 10x Genomics Visium protocol. (**B**) Clinical characteristics of the cohort, including tumor origin, stage, grade, treatment status, and availability of multiomic data after quality control. (**C**) UMAP embeddings of snRNA-seq and snATAC-seq data colored by annotated cell types. Bar plots indicate the relative abundance of major cell populations across samples.

**Figure 2:**
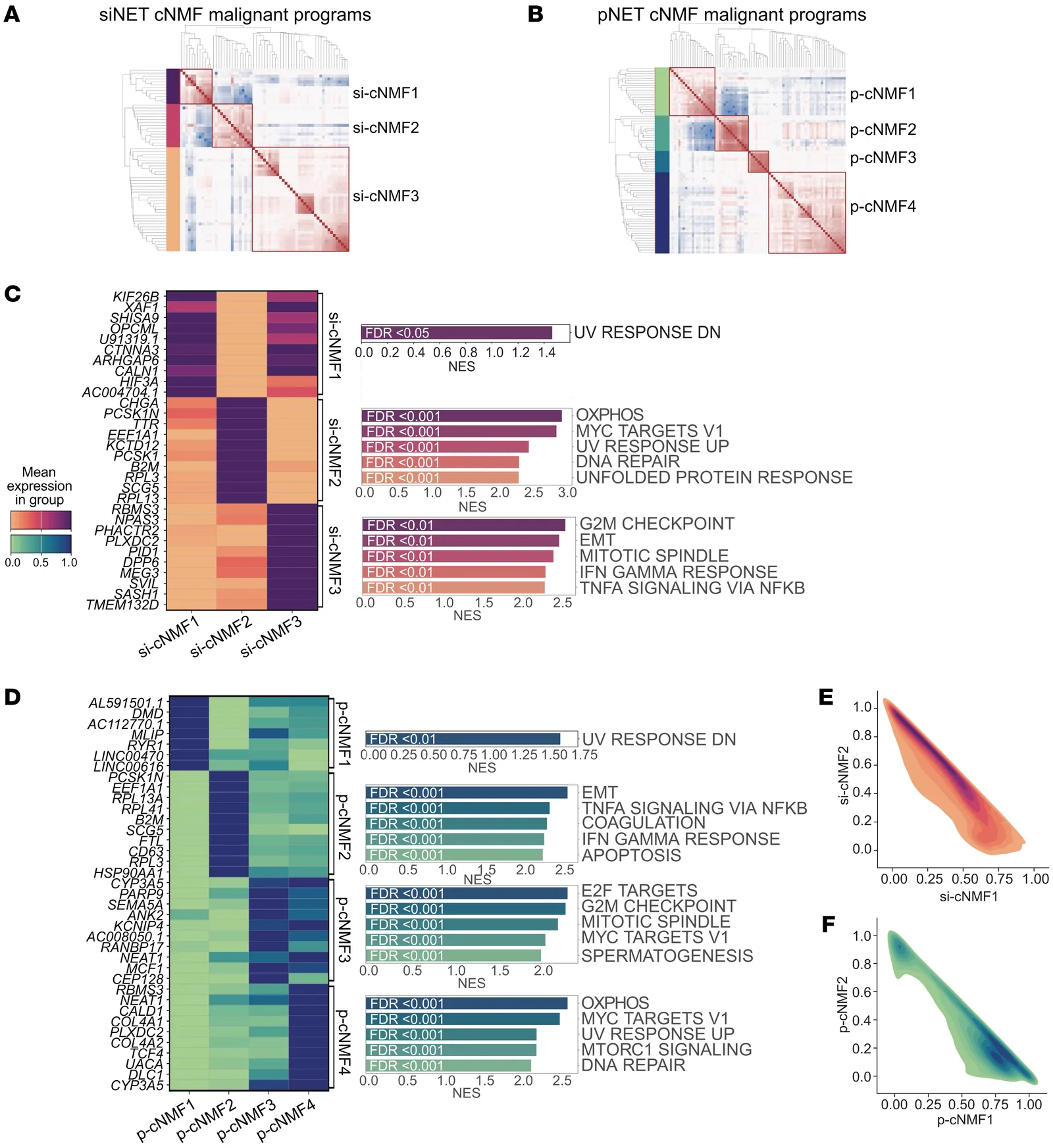
Identification and characterization of malignant cNMF programs in siNETs and PanNETs. (**A** and **B**) Heatmaps showing gene weights for cNMF programs identified independently in malignant cells from siNETs (**A**) and PanNETs (**B**). (**C** and **D**) GSEA of hallmark pathways associated with each cNMF program. Normalized enrichment scores (NESs) and FDR-adjusted *q* values are shown. (**E** and **F**) 2D density plots of normalized neuronal-like (cNMF1) versus secretory neuroendocrine (cNMF2) program scores in siNETs (**E**) and PanNETs (**F**), illustrating a continuous transcriptional spectrum.

**Figure 3:**
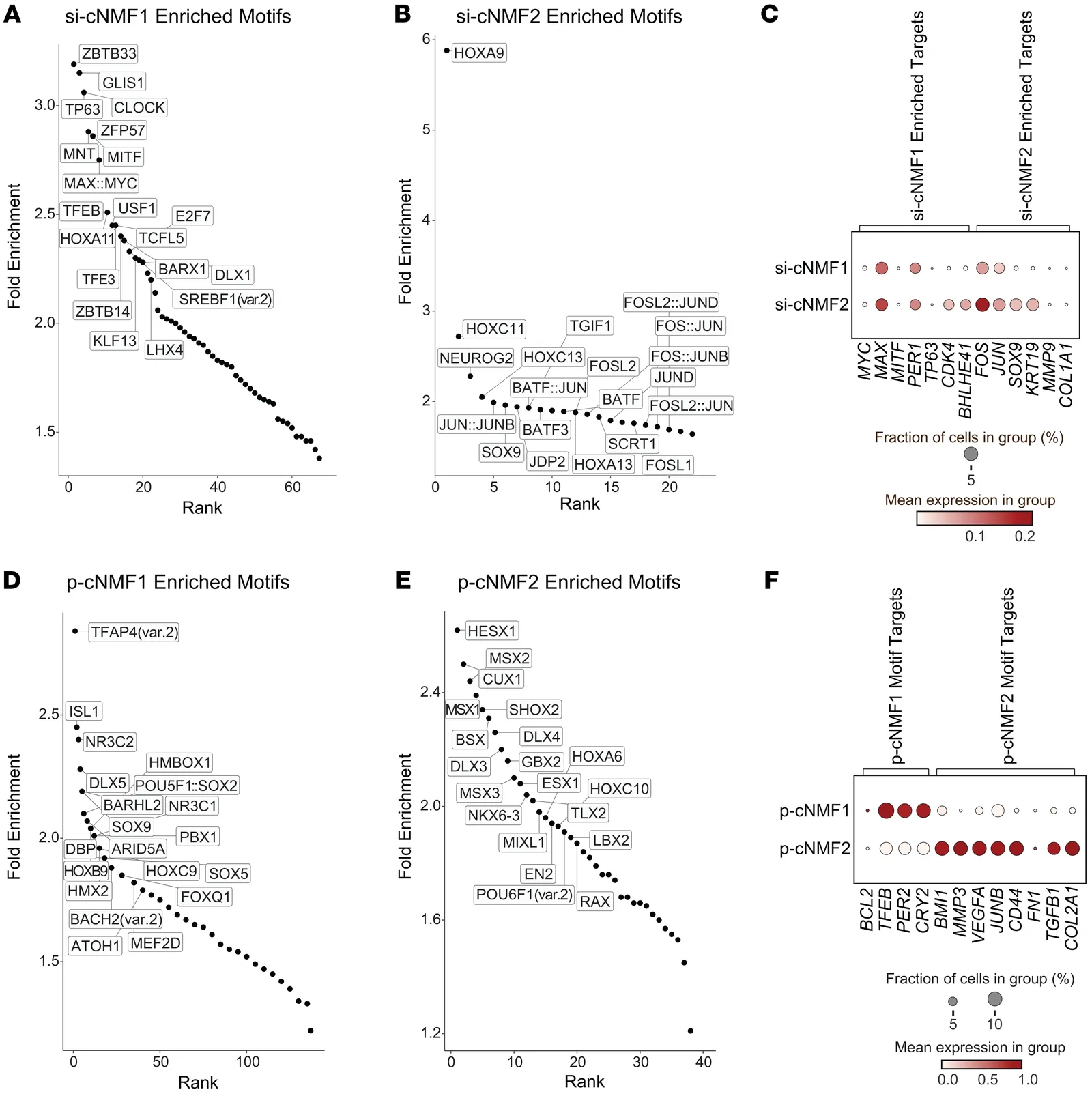
Distinct epigenetic landscapes regulate conserved malignant transcriptional programs in siNETs and PanNETs. (**A** and **B**) TF binding motif enrichment analysis of snATAC-seq peaks associated with siNET malignant cells enriched for the neuronal-like cNMF1 program (**A**) and secretory cNMF2 program (**B**). Motif enrichment significance was assessed using chromVAR deviation scores. (**C**) Expression of predicted target genes corresponding to enriched TF motifs in siNET malignant cells. (**D** and **E**) TF motif enrichment for PanNET malignant cells enriched for neuronal-like p-cNMF1 (**D**) and secretory p-cNMF2 (**E**) programs. (**F**) Expression of predicted target genes for PanNET-enriched motifs. Statistical testing was performed as described in Methods.

**Figure 4:**
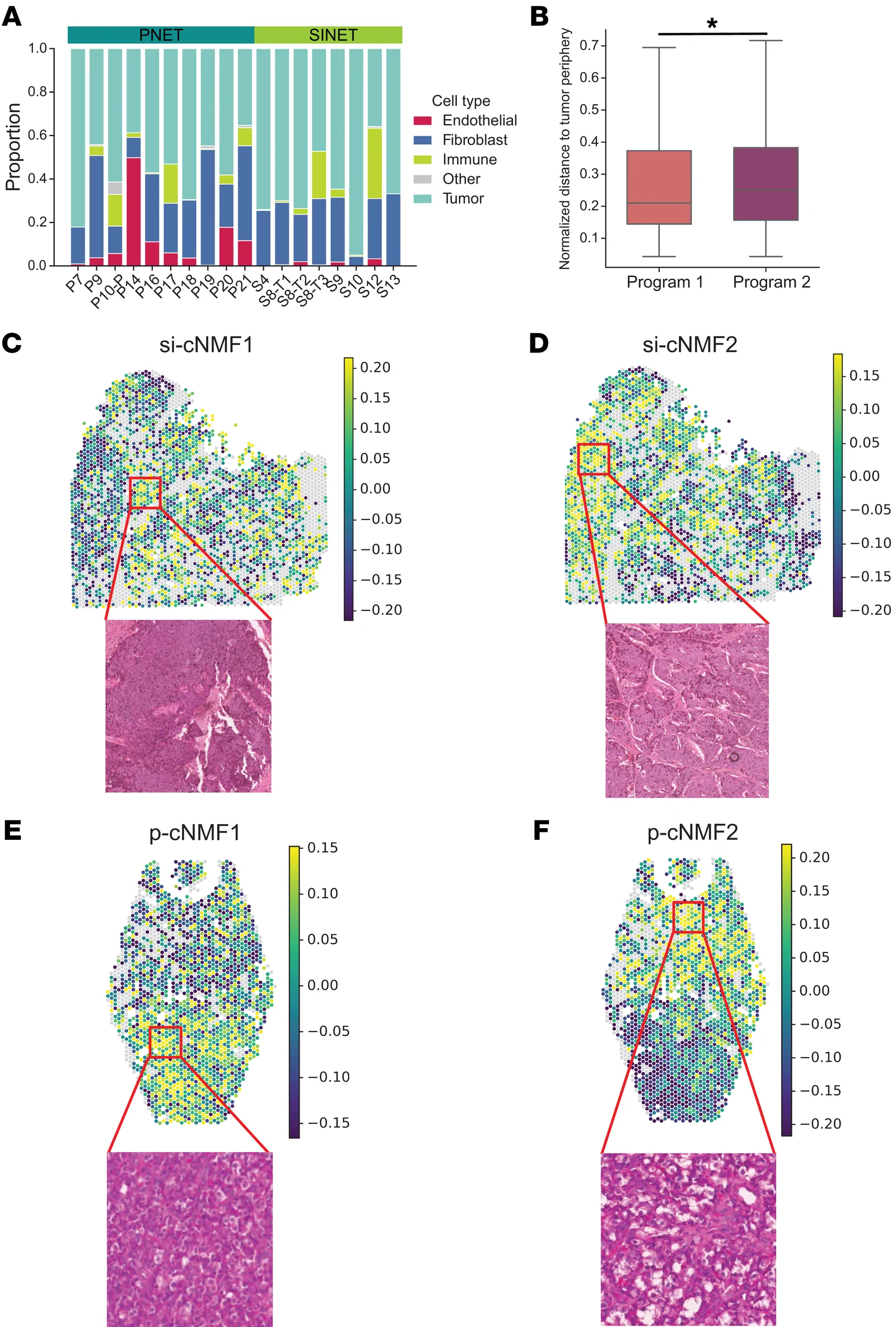
Spatial transcriptomics reveals distinct localization of malignant cNMF programs within tumors. (**A**) Proportions of inferred malignant, stromal, immune, and normal tissue regions across spatial transcriptomic samples. (**B**) Distance of malignant spots enriched for cNMF1 or cNMF2 from the tumor boundary. Distances were compared using a 2-sided Wilcoxon’s rank-sum test. The box-and-whisker plots depict the minimum and maximum values (whiskers), the upper and lower quartiles, and the median. *P < 0.05. (**C** and **D**) Representative siNET spatial maps showing enrichment of cNMF1-high malignant spots in densely cellular regions (**C**) and cNMF2-high malignant spots in stroma-rich regions (**D**). (**E** and **F**) Spatial distribution of PanNET malignant spots enriched for p-cNMF1 (**E**) and p-cNMF2 (**F**). All spatial maps are representative examples.

**Figure 5:**
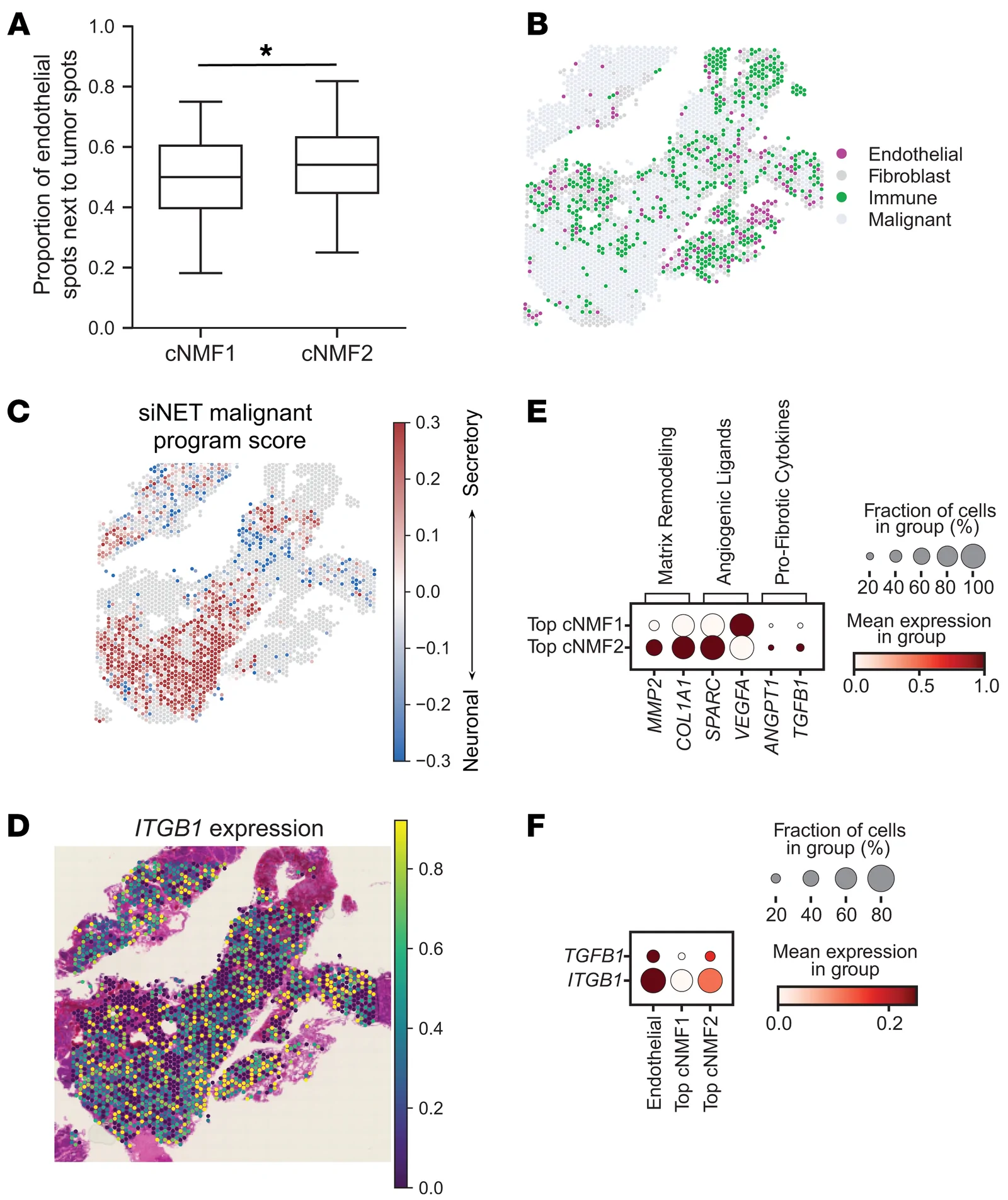
Secretory neuroendocrine program–high malignant cells spatially associate with endothelial cells and exhibit stromal and angiogenic transcriptional programs. (**A**) Fraction of endothelial cells adjacent to cNMF1-high versus cNMF2-high malignant spots across spatial transcriptomic samples. Statistical significance was assessed using a 2-sided Wilcoxon’s signed-rank test across samples. The box-and-whisker plots depict the minimum and maximum values (whiskers), the upper and lower quartiles, and the median. *P < 0.05. (**B** and **C**) Representative PanNET spatial maps showing localization of endothelial cells adjacent to cNMF2-high malignant spots. (**D**) Dot plot showing expression of profibrotic, extracellular matrix remodeling, and angiogenic genes in malignant spots stratified by cNMF program. Per-gene expression differences were assessed using a Wilcoxon’s rank-sum test, with *P* values adjusted for multiple testing using the Benjamini-Hochberg method. (**E** and **F**) Spatial expression of *TGFB1* and *ITGB1*, demonstrating polarized expression in cNMF2-high malignant regions.

**Figure 6:**
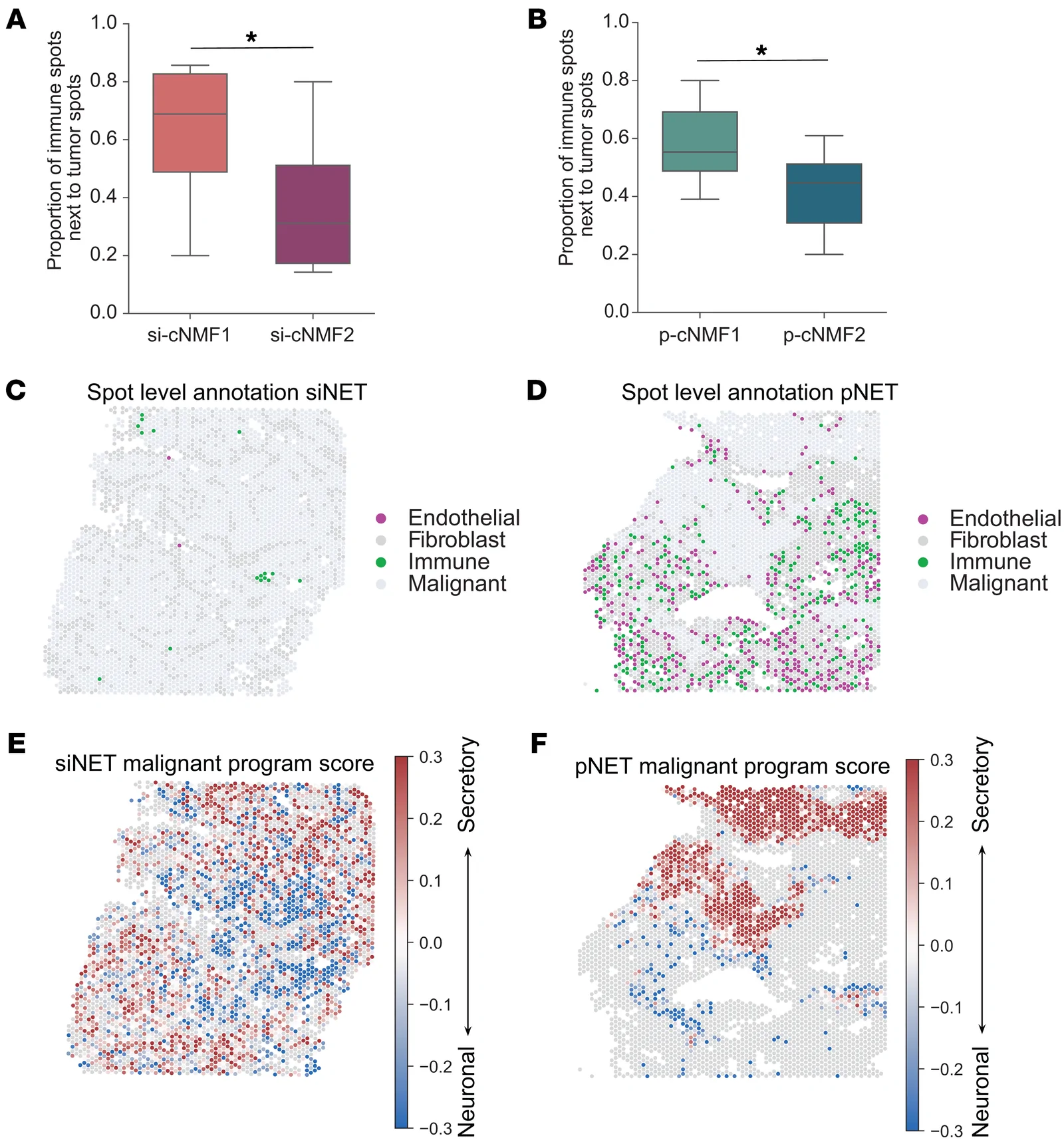
Neuronal-like program–high malignant cells localize to stroma-sparse tumor regions with immune infiltration. (**A** and **B**) Proportion of immune cells adjacent to cNMF1-high versus cNMF2-high malignant spots in siNETs (**A**) and PanNETs (**B**). Differences were assessed using a 2-sided Wilcoxon’s signed-rank test across samples. The box-and-whisker plots depict the minimum and maximum values (whiskers), the upper and lower quartiles, and the median. *P < 0.05. (**C** and **D**) Representative spatial maps showing immune cell localization relative to malignant spots in siNET (**C**) and PanNET (**D**) samples. (**E** and **F**) Spatial distribution of malignant cNMF program scores highlighting immune cell–dense regions enriched for cNMF1-high malignant spots.

**Figure 7:**
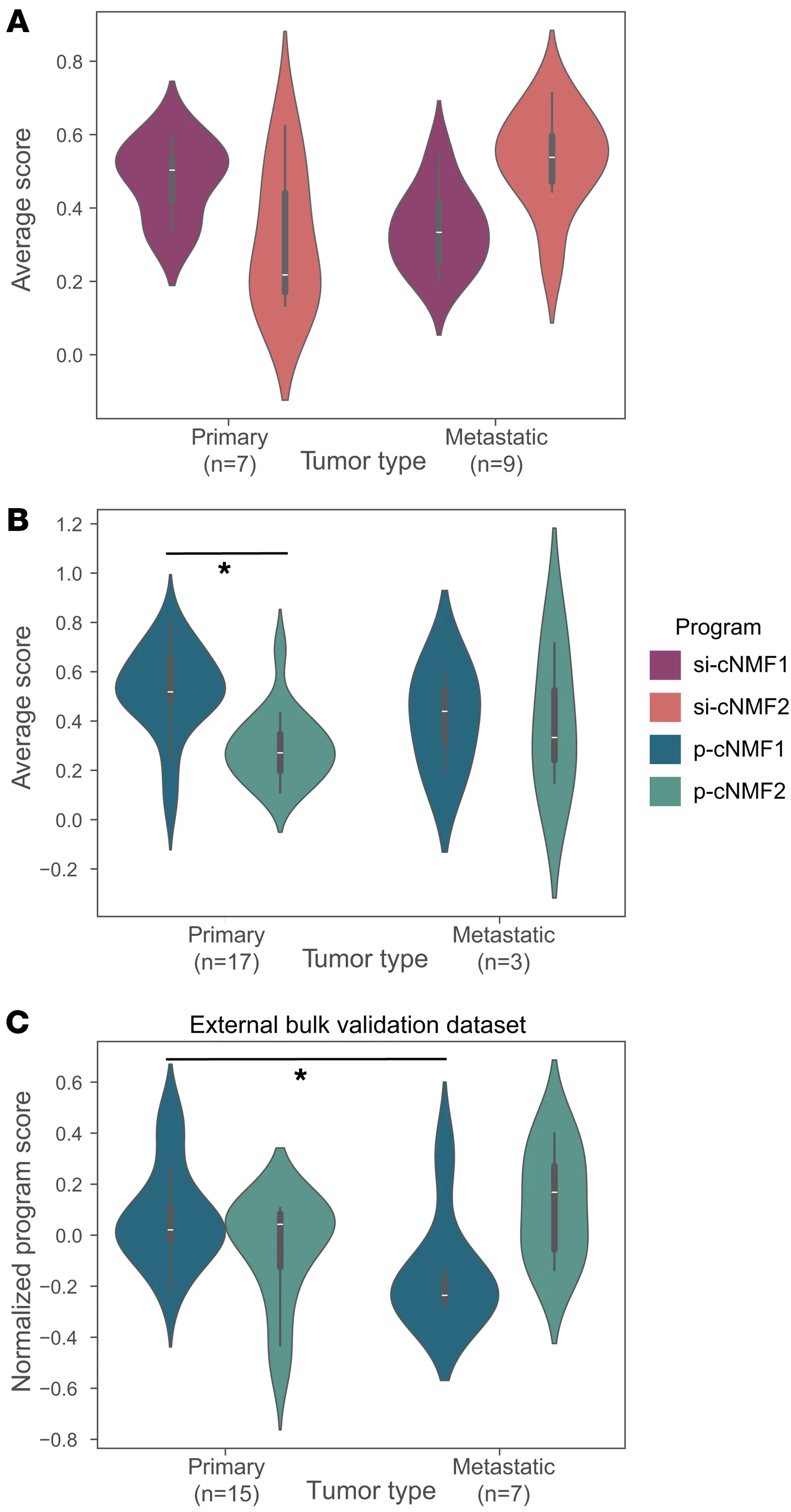
Malignant transcriptional programs associate with clinical stage and metastatic progression. (**A** and **B**) Violin plots showing patient-level mean cNMF program scores derived from malignant snRNA-seq profiles and stratified by primary versus metastatic disease for siNETs (**A**) and PanNETs (**B**). cNMF1 and cNMF2 program scores were compared between primary and metastatic tumors using 2-sided Wilcoxon’s rank-sum tests, while cNMF1 and cNMF2 scores within primary or metastatic tumors were compared using paired 2-sided Wilcoxon’s signed-rank tests. (**C**) Validation of PanNET cNMF program associations in an independent bulk RNA-seq PanNET cohort, in which each sample represents an individual tumor. The same between-group and within-sample comparisons were performed. Within each panel, *P* values for the 4 planned comparisons were adjusted using Holm’s method to control the family-wise error rate. Statistical significance is based on Holm-adjusted *P* values. *P < 0.05.

## Version 1 09/01/2026

In-Press Preview

## Version 2 11/02/2026

Electronic publication
