# Supplementary material for: Conserved neuronal-like and secretory programs define the spatial architecture of gastroenteropancreatic neuroendocrine tumors
Source: J Clin Invest. 2026 Sep 1;136(21):e204119. doi: 10.1172/JCI204119 (PMC13722396; doi:10.1172/JCI204119)
Supplement: Supplementary file 1 [file jci-136-204119-s066.pdf]

METHODS

Xenium ST data preparation

Formalin-fixed, paraffin-embedded (FFPE) tissue sections (5 µm) from four patients were placed on Xenium slides following the demonstrated protocol guide (CG000578). Deparaffinization and tissue permeabilization were performed according to the 10x Genomics Xenium User Guide (GC000580). Probe hybridization and cell segmentation staining were carried out following the 10x Genomics User Guide (CG000760 Rev B). The hybridized slide was then loaded onto the Xenium Analyzer in accordance with the User Guide (CG000584 Rev G). The 5K Prime Human Pan-Tissue & Pathway Panel was used for this study. Post-Xenium Analyzer run H&E staining was performed according to the demonstrated protocol CG000613.

Xenium ST data preprocessing and cell type annotation

Xenium spatial transcriptomics data were preprocessed using the SOPA software,134 following established guidelines. Initial cell segmentation was performed using the Cellpose135 “cyto3” model with parameters recommended in the SOPA tutorial, using the DAPI and ATP1A1/CD45/E-Cadherin channels for detection. For each sample, cells with fewer than 10 counts were excluded from downstream analysis. Cell type annotation was conducted through Leiden clustering on a 15-nearest neighbor graph constructed from a 50-component PCA. Clusters were manually annotated based on canonical marker analysis and comparison with single-nuclei population markers to ensure accurate cell type classification.

Xenium ST data analysis and program correlation

To conservatively project single-cell–derived malignant transcriptional programs onto Xenium data, we first identified genes shared between the snRNA-seq–derived cNMF programs and the Xenium gene panel. Limited overlap was observed between the cNMF programs and available Xenium targets, with 3-15 genes represented per program. These overlapping genes

were used to score malignant Xenium cells using AUCell. Program activity scores were calculated for each malignant cell, and cells within the top decile (90th percentile) of a given program score were classified as program-high. To increase specificity of program assignment, “exclusive” program-high malignant cells were defined as cells within the top decile of one cNMF program while excluding cells simultaneously classified as high for the opposing malignant program. Downstream analyses were restricted to confidently annotated malignant cells.

Spatial proximity analyses were performed to assess relationships between malignant transcriptional states and stromal or immune populations. For each malignant program, nearest-neighbor distances to the indicated cell populations were calculated. Relative proximity values were defined as the difference between the median distances of program-low and program-high malignant cells to the indicated stromal or immune population, such that positive values indicate that program-high malignant cells are spatially closer to the specified cell type.

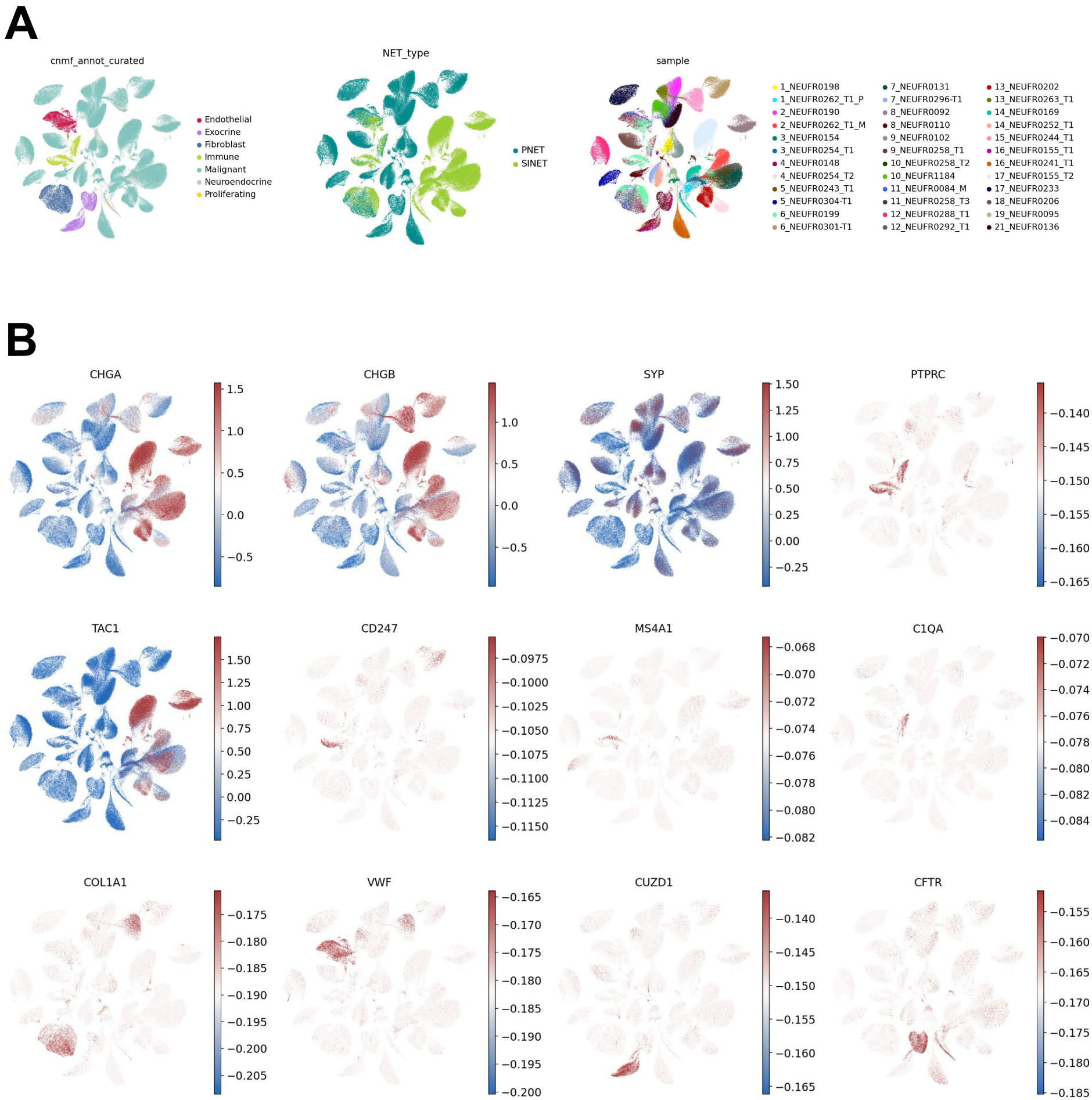

Supplemental Figure 1: Cell type annotation and malignant cell identification in single-nucleus multi-omic data. (A) UMAP embedding of snRNA-seq data colored by broad cell type annotations derived from Leiden clustering and canonical marker gene expression, including malignant cells (*CHGA, CHGB, SYP, TAC1*), immune populations (*PTPRC, CD247, MS4A1, C1QA*), endothelial cells (*VWF*), cancer-associated fibroblasts (CAFs) (*COL1A1*), and normal epithelial and exocrine cells (*CUZD1* and *CFTR*). **(B)** Identification of malignant cells using inferred copy number alteration (CNA) profiles combined with expression of established neuroendocrine tumor marker genes. Heatmaps display large-scale chromosomal gains and losses across malignant and non-malignant populations. CNA inference was performed as described in Methods and used to distinguish malignant tumor cells from surrounding stromal and immune cells.

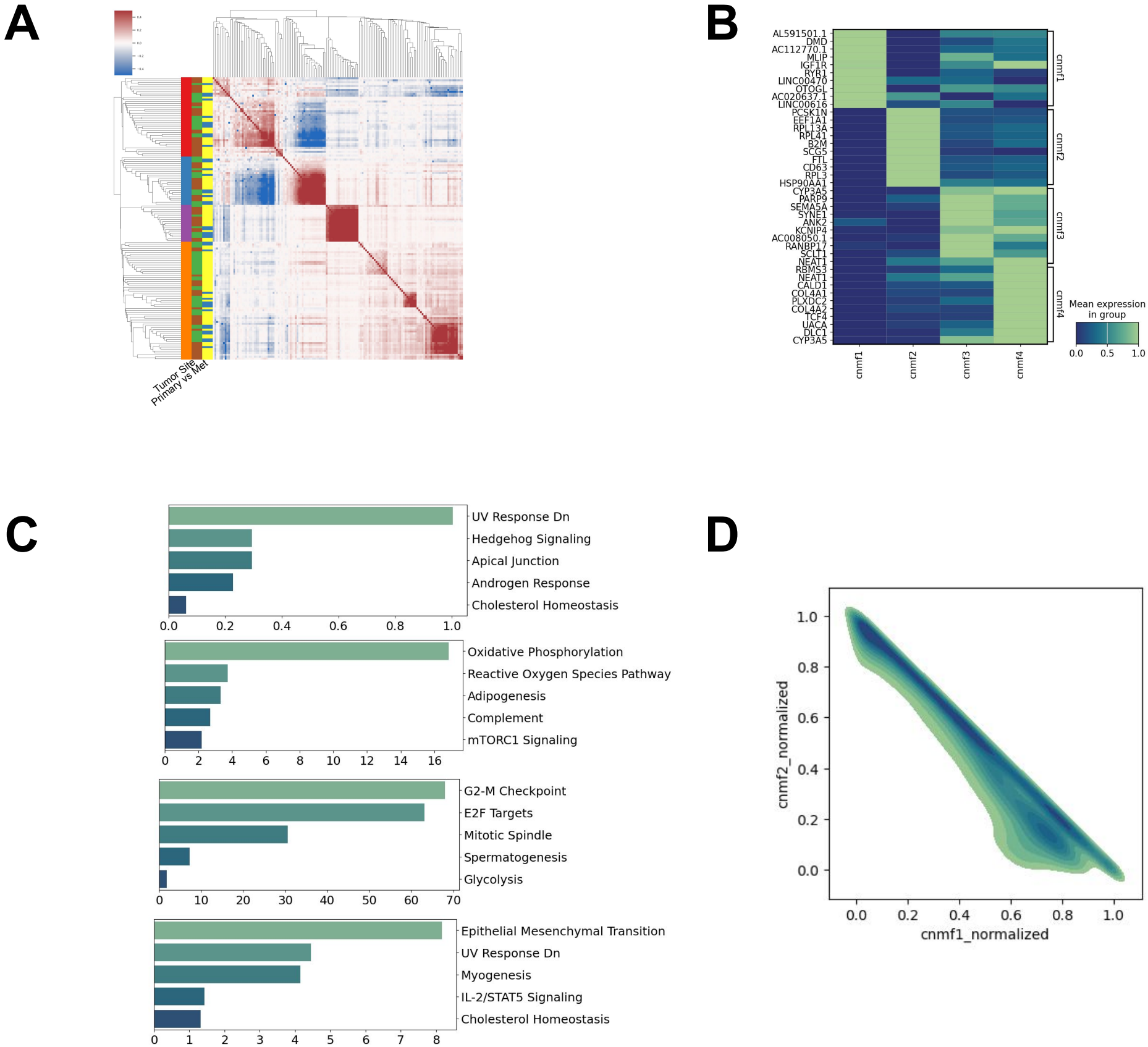

Supplemental Figure 2: Integrated cNMF analysis reveals conserved malignant transcriptional programs across tumor types. Integrated consensus non-negative matrix factorization (cNMF) was performed on pooled malignant cells from siNETs and pNETs. **(A)** Heatmap of gene weights for integrated cNMF programs, demonstrating overlap with programs identified independently in siNETs and pNETs. The programs are also independent of primary vs metastatic samples. **(B)** Contribution of siNET- and pNET-derived malignant cells to each integrated cNMF program, illustrating that both tumor types contribute to neuronal-like and secretory neuroendocrine programs. **(C)** Gene set enrichment analysis (GSEA) of hallmark pathways associated with each cNMF program. **(D)** Density plots of normalized integrated cNMF program scores showing continuous distributions rather than discrete subpopulations. These analyses support the presence of conserved malignant cell states across organ sites.

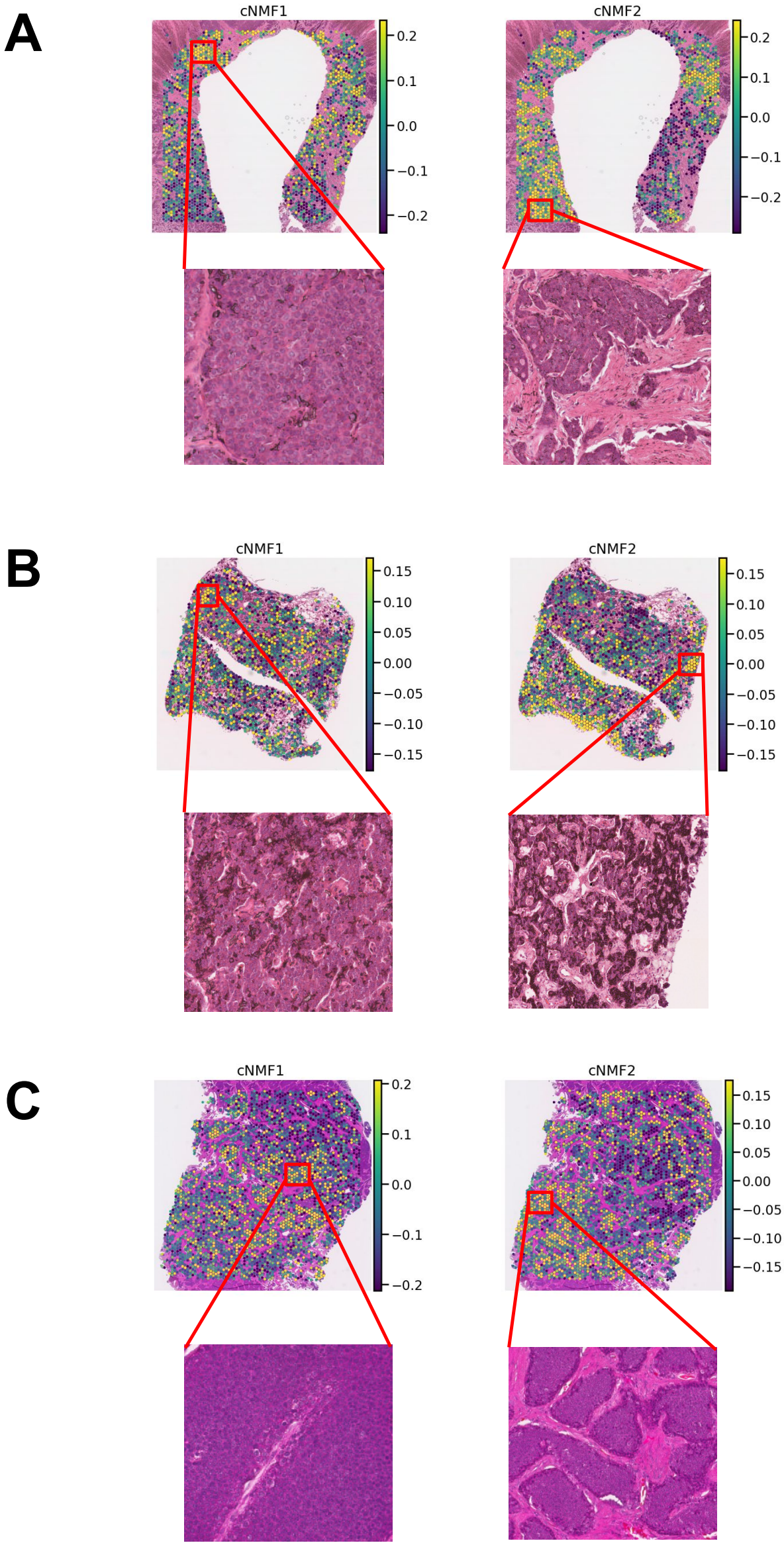
A
cNMF1
0.2
0.1
0.0
−0.1
−0.2
cNMF2
0.2
0.1
0.0
−0.1
−0.2
B
cNMF1
0.15
0.10
0.05
0.00
−0.05
−0.10
−0.15
cNMF2
0.15
0.10
0.05
0.00
−0.05
−0.10
−0.15
C
cNMF1
0.2
0.1
0.0
−0.1
−0.2
cNMF2
0.15
0.10
0.05
0.00
−0.05
−0.10
−0.15

Supplemental Figure 3: Additional spatial examples validating localization of malignant cNMF programs. cNMF1 and cNMF2 scoring of malignant spots on spatial transcriptomics data with representative spatial transcriptomic sections demonstrating localization of cNMF1-high malignant spots to densely cellular tumor regions and cNMF2-high malignant spots to stromal-infiltrated regions. **(A)** sample S13, **(B)** sample S8-T2, and **(C)** sample S4. Across samples, spatial enrichment patterns were consistent with those shown in Figure 4. These panels provide qualitative validation across multiple tumors; statistical analyses of spatial proximity and boundary distance are presented in the main figures.

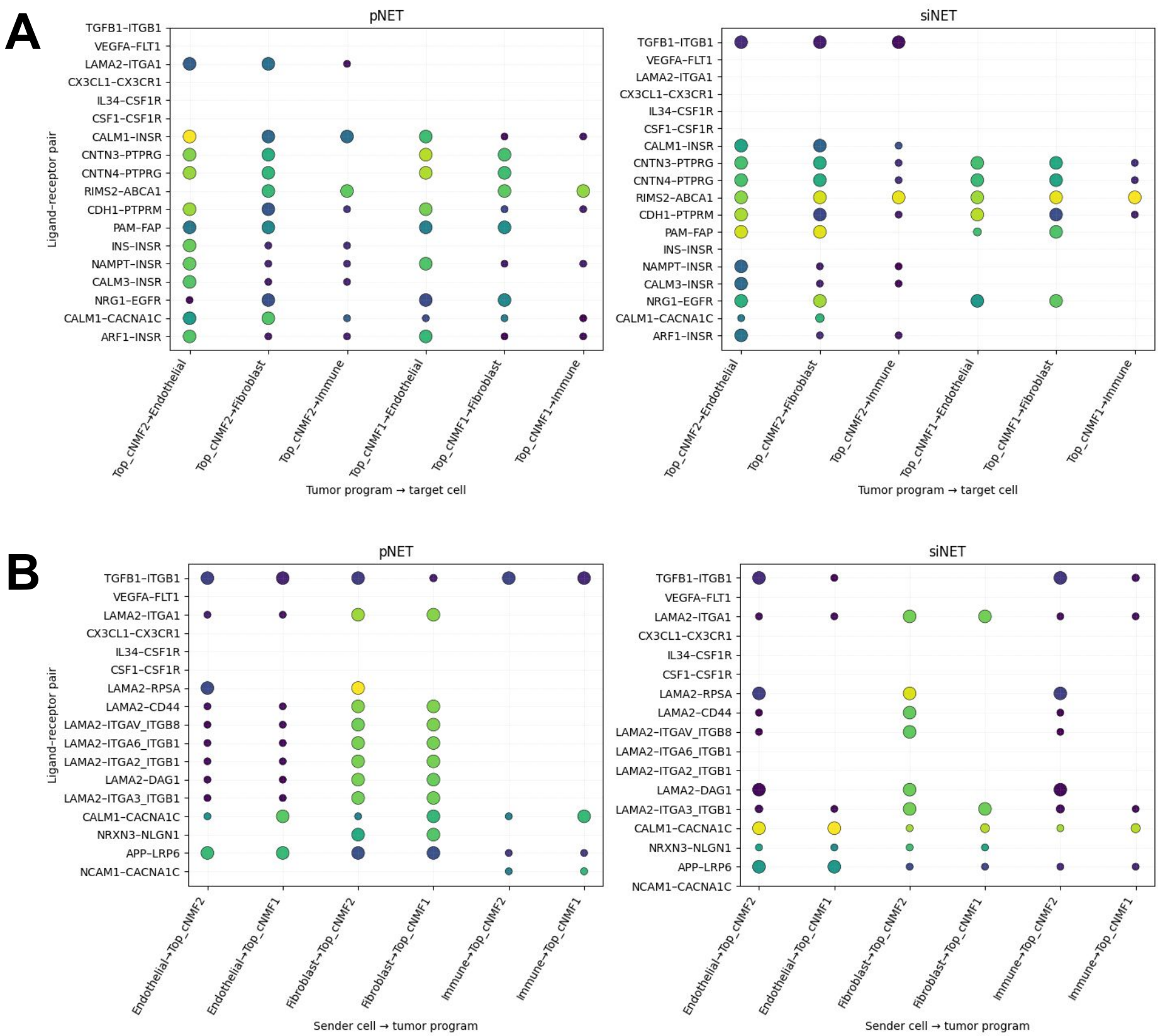

Supplemental Figure 4: Bidirectional ligand–receptor signaling associated with malignant cell programs. Bubble plots show prioritized ligand–receptor interactions involving Top_cNMF1 and Top_cNMF2 malignant cell programs in pancreatic and small intestinal neuroendocrine tumors (pNETs and siNETs). **(A)** Outgoing signaling from malignant programs to stromal and immune populations reveals enriched endothelial and fibroblast interactions from Top_cNMF2 malignant cells, whereas Top_cNMF1 malignant cells preferentially engage immune and myeloid populations. **(B)** Incoming signaling from stromal and immune populations to malignant programs demonstrates reciprocal patterns, with Top_cNMF2 malignant cells receiving prominent vascular and stromal signals and Top_cNMF1 malignant cells receiving immune- and neuronal-associated signals. Dot size represents interaction significance (−log10 p-value), and dot color represents interaction strength as quantified by LIANA.

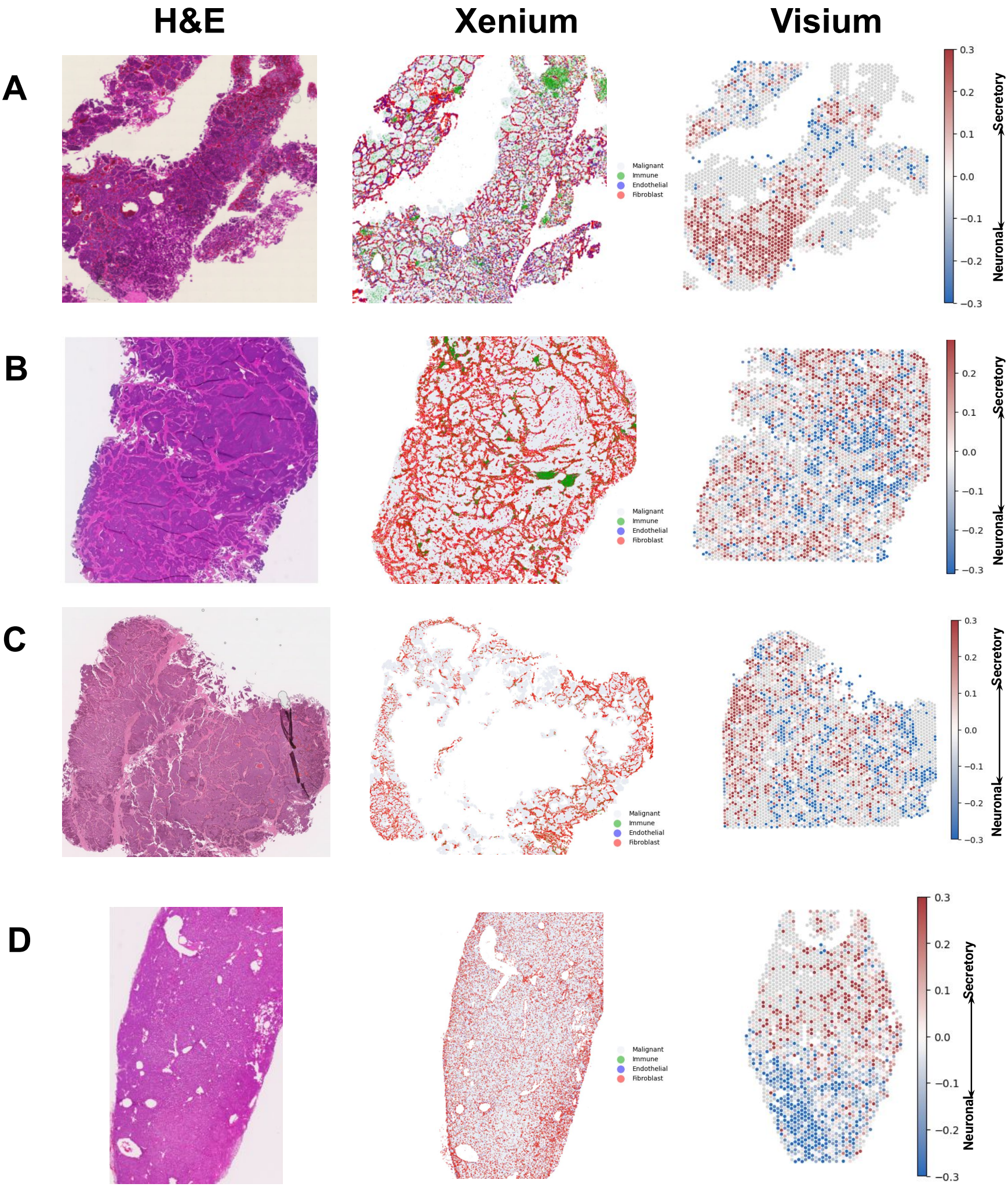
H&E
Xenium
Visium
A
B
C
D
Malignant
Immune
Endothelial
Fibroblast
Secretory
Neuronal
0.3
0.2
0.1
0.0
−0.1
−0.2
−0.3

Supplemental Figure 5: Orthogonal Xenium spatial profiling supports stromal organization associated with Visium-defined malignant programs in GEP-NETs. Representative matched H&E, Xenium, and Visium spatial profiles from four GEP-NET tumors: **(A)** sample P18, **(B)** sample S4, **(C)** sample P9, and **(D)** sample P17. Left panels show corresponding H&E sections. Middle panels show Xenium-derived single-cell spatial annotations, including malignant cells (light gray), fibroblasts (red), endothelial cells (blue), and immune populations (green). Right panels show Visium spatial mapping of relative malignant program enrichment, displayed as cNMF2-biased versus cNMF1-biased regions derived from the snRNA-seq-defined malignant cNMF programs. Red regions indicate relative enrichment of the cNMF2 malignant program, whereas blue regions indicate relative enrichment of the cNMF1 malignant program.

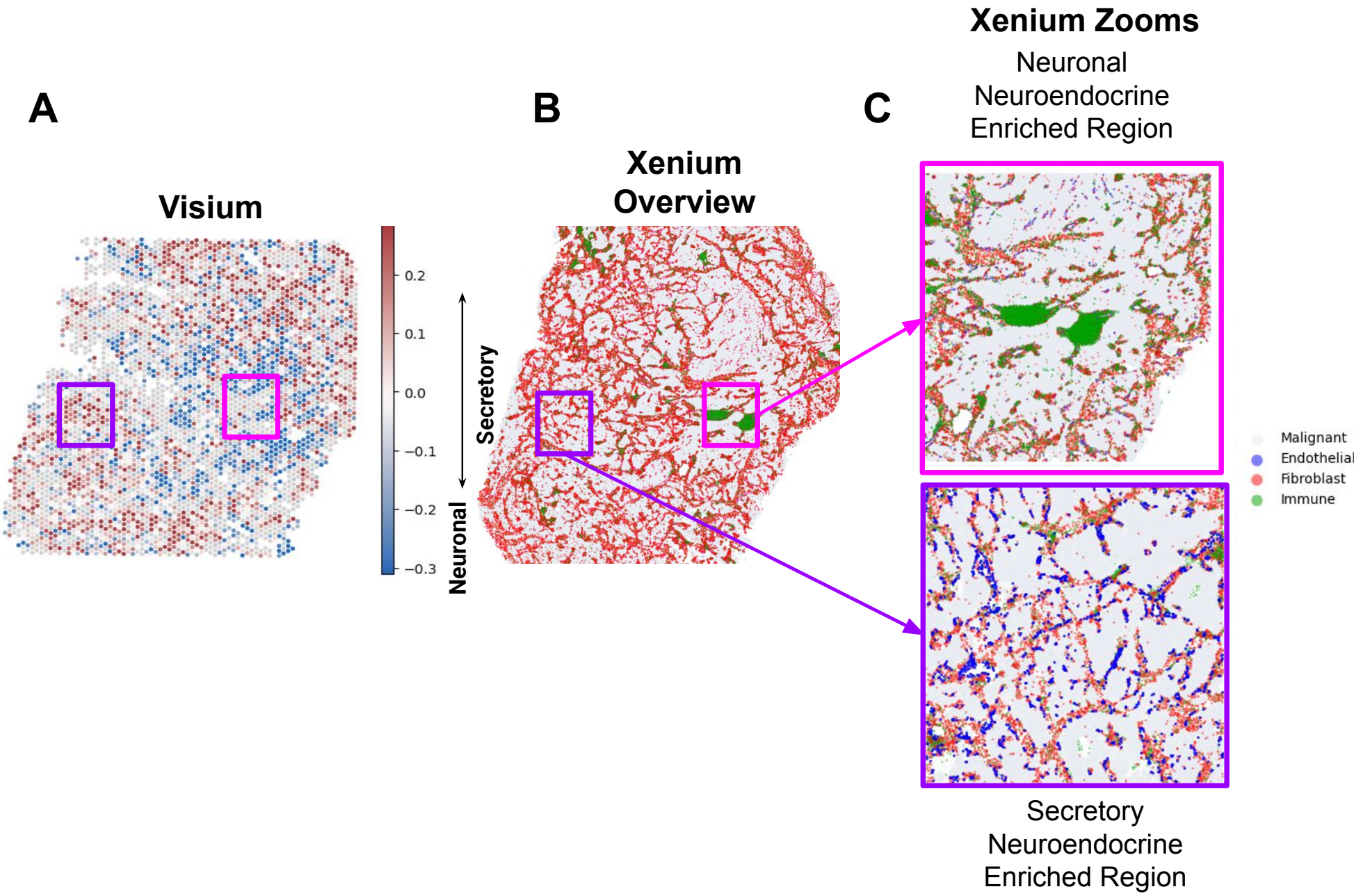

Supplemental Figure 6: Xenium spatial profiling demonstrates distinct stromal and immune microenvironments associated with Visium-defined malignant regions. (A) Visium spatial transcriptomic map showing relative malignant program enrichment within a representative GEP-NET tumor section, displayed as the relative cNMF2 (secretory-like) versus cNMF1 (neuronal-like) score derived from the snRNA-seq-defined malignant programs. Red regions indicate relative cNMF2 enrichment, whereas blue regions indicate relative cNMF1 enrichment. Purple boxes indicate representative regions selected for higher-resolution spatial comparison. **(B)** Corresponding Xenium single-cell spatial annotation from a matched tumor specimen. Cell populations include malignant cells (light gray), fibroblasts (red), endothelial cells (blue), and immune cells (green). The same spatial regions highlighted in panel A are outlined for comparison. **(C)** High-magnification Xenium views of representative Visium-defined niches. The neuronal-like tumor region displays stromal depletion and immune enrichment. The secretory neuroendocrine enriched tumor region demonstrates organized fibrovascular stromal architecture enriched for fibroblast and endothelial populations. Together, these findings provide orthogonal single-cell-resolution support for the regional stromal and immune microenvironmental organization inferred from the Visium analyses.

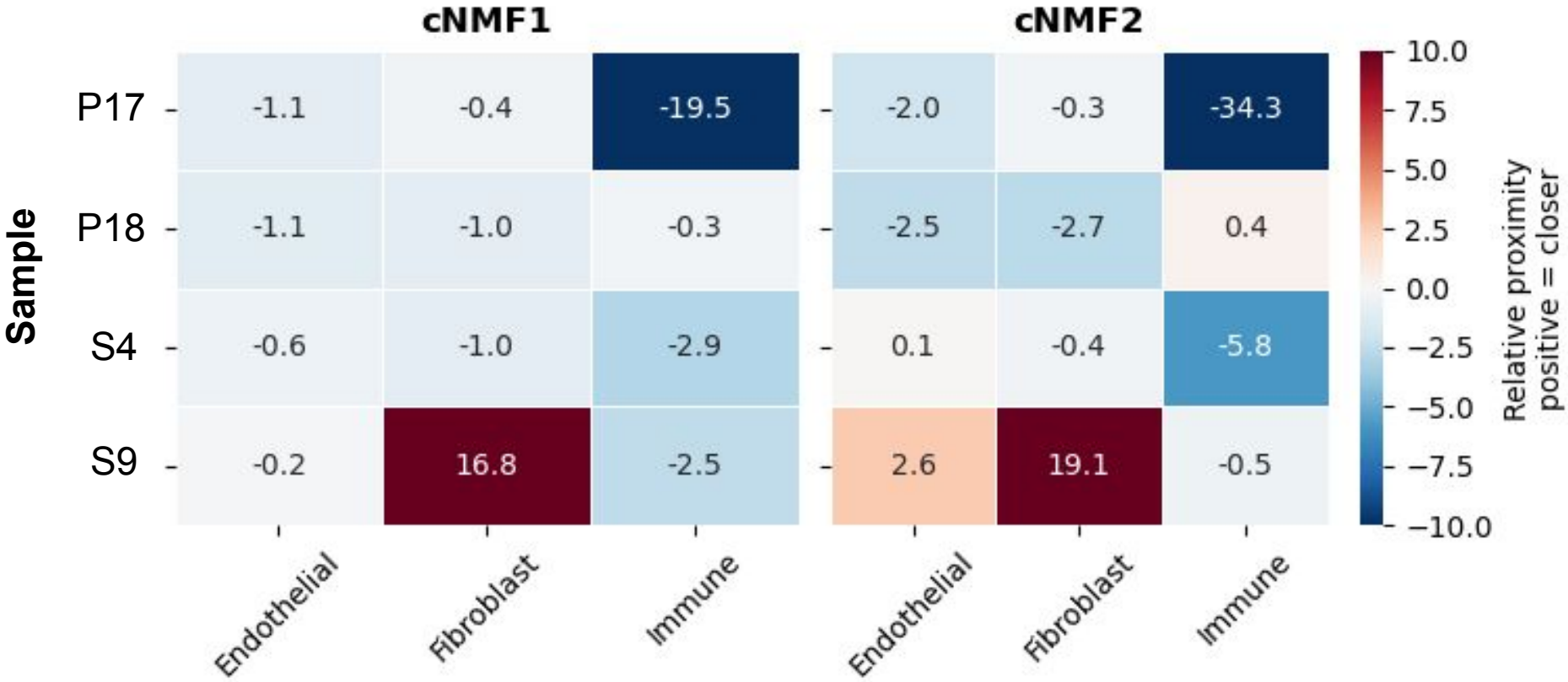

Supplemental Figure 7: Quantitative Xenium spatial analysis of stromal and immune populations associated with Visium-defined malignant programs. Heatmaps showing the relative spatial proximity of exclusive cNMF1-high and cNMF2-high malignant cells to endothelial, fibroblast, and immune populations across Xenium-profiled tumors. Representative malignant program scores were calculated using overlapping Xenium genes derived from the Visium-defined cNMF programs. Program-high malignant cells were defined as cells within the top decile for one program while excluding cells simultaneously classified as high for the opposing program ("exclusive" program-high cells). Analyses were restricted to confidently annotated malignant cells. Relative proximity values represent the difference between median distances of program-low and program-high malignant cells to the indicated stromal or immune population, such that positive values indicate that program-high malignant cells are spatially closer to the indicated cell population. Across representative samples, cNMF2-high malignant cells demonstrated preferential proximity to fibroblast-rich stromal regions and, in some samples, endothelial-associated niches, consistent with the stromal remodeling and fibrovascular features identified in the Visium analyses. In contrast, cNMF1-high malignant cells generally demonstrated comparatively reduced association with stromal compartments.

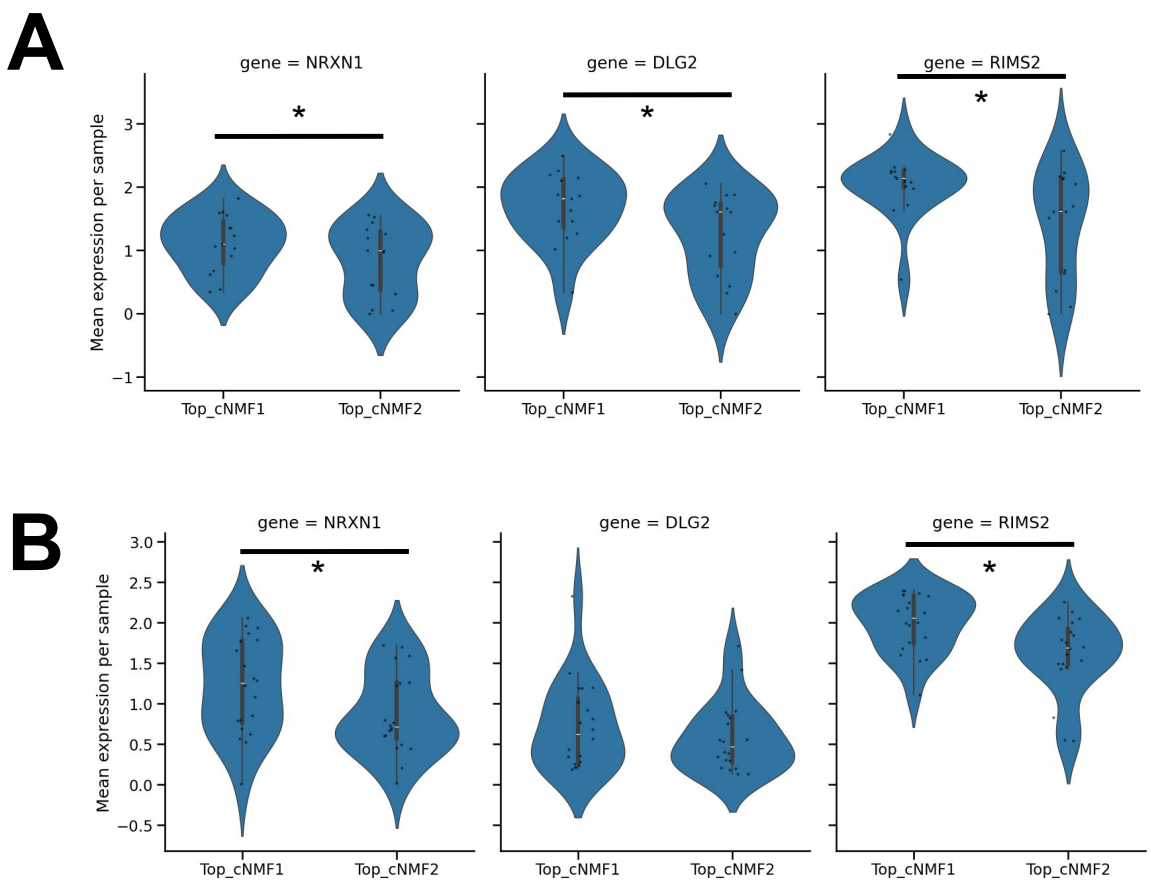

Supplementary Figure 8: Neuronal communication–associated genes are enriched in neuronal-like-high malignant cells. Violin plots show per-sample mean expression of the neuronal communication–associated genes *NRXN1, DLG2,* and *RIMS2* in malignant cells stratified by Top-cNMF program (Top-cNMF1 vs. Top-cNMF2). **(A)** siNET samples were scored for si-cNMF1 amd si-cNMF2. **(B)** PanNET samples were scored for p-cNMF1 and p-cNMF2. For each sample, gene expression was averaged across all malignant cells assigned to the indicated Top-cNMF program. Black points represent individual samples. Statistical significance was assessed using a paired two-sided Wilcoxon signed-rank test across samples, with p-values adjusted for multiple testing using the Benjamini–Hochberg method. * = p < 0.001

A

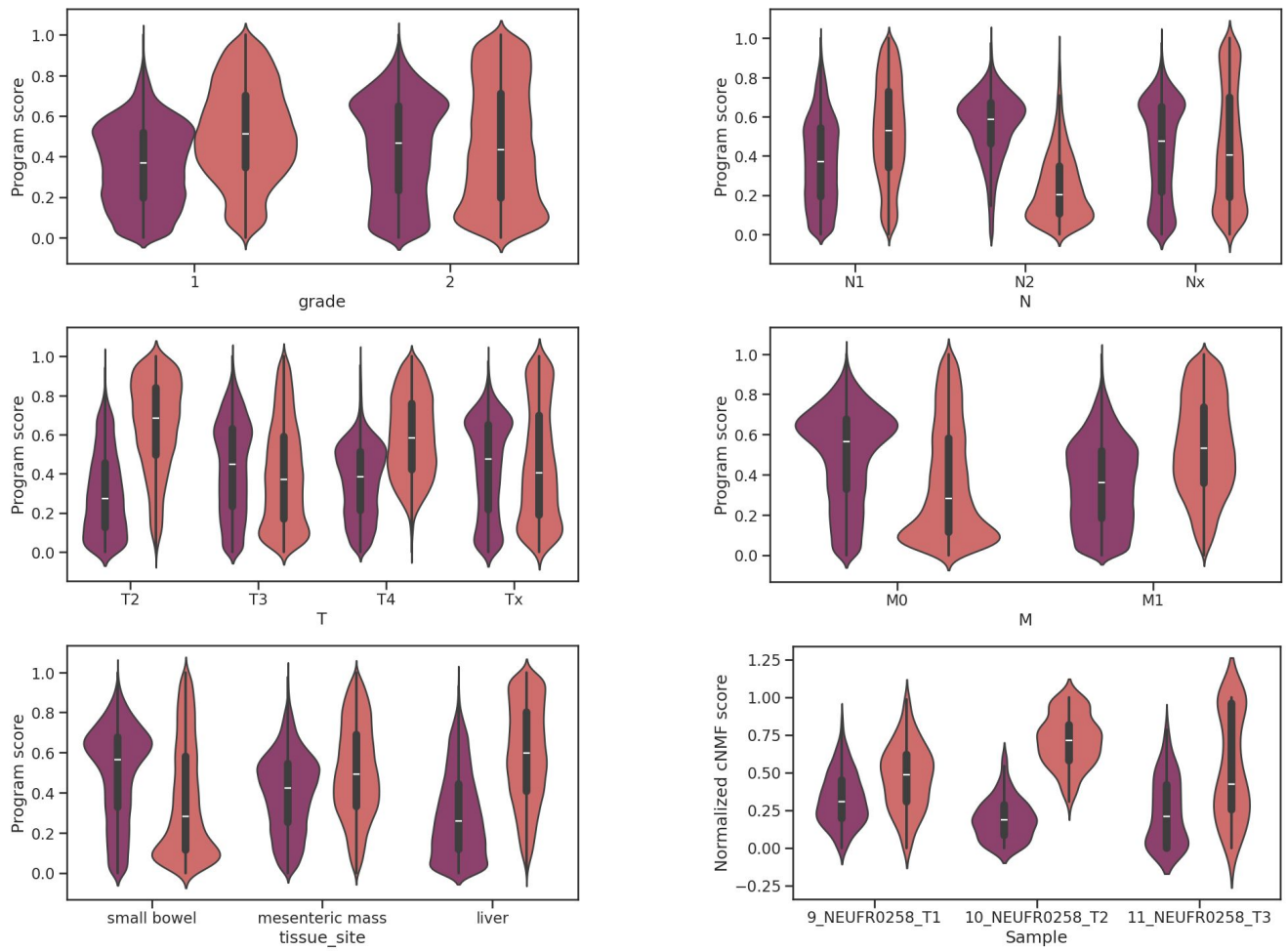

Program score
grade
1
2
N
N1
N2
Nx
T
T2
T3
T4
Tx
M
M0
M1
tissue_site
small bowel
mesenteric mass
liver
Normalized cNMF score
Sample
9_NEUFR0258_T1
10_NEUFR0258_T2
11_NEUFR0258_T3

B

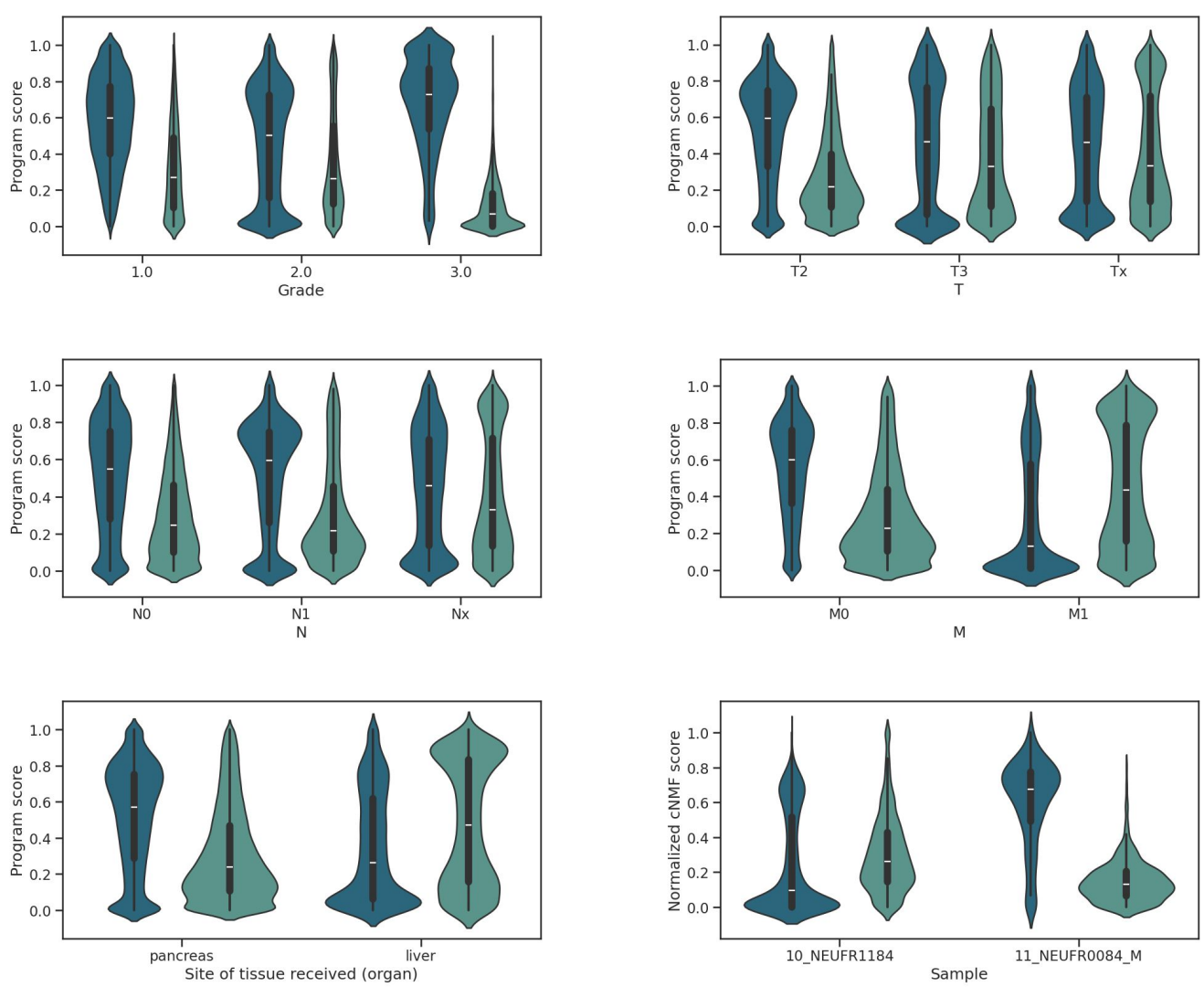

Program score
Grade
1.0
2.0
3.0
T
T2
T3
Tx
N
N0
N1
Nx
M
M0
M1
Site of tissue received (organ)
pancreas
liver
Normalized cNMF score
Sample
10_NEUFR1184
11_NEUFR0084_M

Supplementary Figure 9: Malignant cNMF program scores associate with metastatic status and progression. (A) Distribution of si-cNMF program scores stratified by Grade, N, T, M, and tissue site across siNETs. Within-patient analysis of a representative siNET case (sample S8) sampled across primary, regional metastatic, and distant metastatic lesions. Statistical significance was assessed using a two-sided Wilcoxon rank-sum test across samples. **(B)** Distribution of p-cNMF program scores stratified by Grade, N, T, M, and tissue site across PanNETs. Statistical significance was assessed using a two-sided Wilcoxon rank-sum test across samples.
